# A comparative meta-analysis of seven types of exercise-based physical therapy for gait stabilization, fall risk, and postural control in Parkinson’s disease patients

**DOI:** 10.3389/fneur.2025.1706561

**Published:** 2025-12-05

**Authors:** Guojun Tong, Juan Ouyang, Yi Xia, Xianjie Cai

**Affiliations:** 1 Chengdu Sport University, Chengdu, China; 2 Shanghai University of Sport, Shanghai, China

**Keywords:** Parkinson’s disease, exercise intervention, gait stabilization, fall risk, postural control

## Abstract

**Background:**

Patients with Parkinson’s disease (PD) often experience impaired gait stabilization, increased risk of falls, and postural control disorders. Although drug and surgical treatments can partially alleviate symptoms, non-pharmacological interventions (such as physical therapy) have become a research focus due to their safety and multi-system benefits. This study used a network meta-analysis to compare the efficacy of seven types of physical therapy in improving motor dysfunction in PD patients.

**Methods:**

The system searched databases such as PubMed, Web of Science, Embase, the Cochrane Library, and China National Knowledge Infrastructure (CNKI) to collect relevant randomized controlled trials (RCTs) published between 2008 and 2024. The study included seven different types of exercise intervention therapies, including RPT, AE, PCT, MBET, TCRT, SSMT, and RTRT. The primary outcome measures included gait stabilization, fall risk, and postural control. Statistical analysis was performed using Stata 18.0 software, with effect sizes combined using a random-effects model. The effects of different exercise interventions were assessed using a network meta-analysis model.

**Results:**

In terms of gait stabilization, MBET ranked highest (SUCRA = 83.1%), but a direct comparison with RPT showed an SMD of −0.32 (95% CI: −0.83 to 0.19, *p* > 0.05), with no statistically significant difference. TCRT was significantly superior to RPT (SMD = −2.42, 95% CI: −3.79 to −1.04, *p* < 0.05), with a SUCRA of 69.0%. There was no significant difference between SSMT and RPT (SMD = −0.48, 95% CI: −1.03 to 0.07, *p* > 0.05), but SUCRA ranked third (60.4%). The Egger test indicated moderate bias (*p* = 0.020), potentially overestimating the effect size by 15–20%. For fall risk: RPT was the most effective (SUCRA = 97.4%) and significantly superior to RTRT (SMD = 1.11, 95% CI: 0.66–1.57, *p* < 0.05), MBET ranked second (SUCRA = 69.7%), and was significantly more effective than PCT (SMD = −6.14, 95% CI: −7.86 to −4.42, *p* < 0.05). AE ranked third with SUCRA = 52.2%, showing a significant difference compared to TCRT (SMD = 0.40, 95% CI: 0.01–0.79, *p* = 0.05). No significant bias was observed (*p* = 0.760). Postural control: PCT was the most effective (SUCRA = 92.4%), significantly superior to RTRT (SMD = 3.90, 95% CI: 1.93 to 5.87, *p* < 0.05). RTRT (resistance training) ranked second (SUCRA = 79.3%), but there was no significant difference compared with MBET (SMD = 0.40, 95% CI: −0.32 to 1.12, *p* > 0.05). RPT (SUCRA = 69.5%), but in direct comparisons, MBET was significantly superior to RPT (SMD = −23.50, 95% CI: −32.69 to −14.31, *p* < 0.05).

**Conclusion:**

There are differences in the efficacy of different exercise interventions: MBET, TCRT, and SSMT are more effective in stabilizing gait; RPT, MBET, and AE are more effective in reducing fall risk; and PCT and RTRT are most effective in improving postural control. Clinicians can select the most appropriate intervention based on patient needs. It should be noted that there is moderate publication bias in gait stabilization. Future studies should expand the scope of their searches and include unpublished data to optimize the quality of evidence.

## 1 Introduction

Parkinson’s disease (PD) is a progressive neurodegenerative disorder characterized by dopaminergic neuron loss in the substantia Nigra (1). The most clinically significant motor manifestations involve postural instability and gait dysfunction, which profoundly impact patients’ functional independence (2). Current epidemiological data demonstrate that 80% of PD patients develop characteristic gait abnormalities including reduced step length (>40% decrease) and freezing episodes, elevating their fall risk to 3–5 times that of age-matched controls (3). Concurrent postural control deficits exacerbate this risk, with patients exhibiting 2–3 times greater postural sway than healthy elderly individuals during static and dynamic balance tasks (4). These motor impairments establish a vicious cycle wherein gait dysfunction precipitates falls, which in turn lead to reduced mobility and accelerated functional decline (2). Longitudinal studies reveal that 60–80% of PD patients experience ≥1 fall annually, with 30–50% resulting in fractures (3). Most alarmingly, the one-year mortality rate following PD-related fractures reaches 25–30% (5). Disease progression worsens these outcomes, with 50–80% of mid-to-late-stage patients developing freezing of gait—a phenomenon that increases annual fall frequency to 3–5 times (6–8 times higher than healthy peers) (6). The consequent fear of falling and activity restriction significantly diminishes quality of life and independence (7).

Current therapeutic approaches show limited efficacy for these axial symptoms. Pharmacological interventions improve gait freezing in only 30% of cases, while prolonged use leads to end-of-dose phenomena in 50% of patients after five years and dyskinesias in 90% after ten years (8). Deep brain stimulation demonstrates inconsistent effects on postural control, may exacerbate freezing episodes, and carries substantial risks including surgical infections (3–5%) and hardware complications (5–10%) (9). Although rehabilitation is routinely recommended, conventional physical therapy programs show marked variability, with meta-analyses reporting up to fivefold differences in treatment effects across studies (10).

Exercise-based interventions have emerged as evidence-supported adjunct therapies. Comprehensive reviews confirm that structured exercise programs reduce fall risk by 30–50% and improve Berg Balance Scale scores by 4–6 points in PD populations (11). Modality-specific benefits have been identified: Traditional Chinese Rehabilitation Training (e.g., Tai Chi) enhances step length by 18–25% through controlled weight-shifting exercises (12), while Mind–Body Exercise Training (e.g., yoga) decreases postural sway area by 20–30% via core stabilization techniques (13). Despite these findings, critical evidence gaps persist regarding the comparative effectiveness of different exercise modalities for specific PD symptoms.

This network meta-analysis evaluates seven established exercise therapies: Routine Physical Training (RPT): Evidence-based standardized motor control rehabilitation (14); Aerobic Training (AE): Cardiorespiratory endurance exercises at 60–70% maximum heart rate (15); Postural Control Training (PCT): Balance rehabilitation incorporating biofeedback technologies (16); Traditional Chinese Rehabilitation (TCRT): Tai Chi and related mind–body practices (17); Mind–Body Exercise (MBET): Yoga/Pilates integrating physical-psychological components (16); Sensory Stimulation Training (SSMT): Movement cueing using external stimuli (18); Resistance Training (RTRT): Progressive strength training for postural musculature. Each modality targets distinct pathophysiological mechanisms: RPT provides fundamental motor skill retraining, AE improves cardiovascular endurance, PCT specifically addresses balance control deficits, and RTRT enhances musculoskeletal support. TCRT and MBET uniquely integrate cognitive-motor components, while SSMT optimizes movement initiation through external cueing. Presently, no high-level evidence exists to guide clinicians in selecting among these approaches for specific patient profiles and symptom constellations.

Our study directly addresses this evidence gap through a rigorous network meta-analysis of randomized controlled trials. By establishing comparative efficacy rankings for these seven exercise therapies across three critical domains (gait stabilization, fall risk reduction, and postural control improvement), we aim to provide empirically validated guidance for personalized PD rehabilitation protocols. These findings will inform clinical decision-making to optimize functional outcomes and mitigate the debilitating consequences of mobility impairment in Parkinson’s disease. This study provides a distinct and novel contribution. Its primary innovation lies in a mechanistic-focused comparison of exercise modalities for fall prevention. Unlike previous work centered on fall rates, we specifically investigate the underlying physiological determinants—gait stability and postural control. Furthermore, we offer greater granularity by deconstructing broad exercise categories into seven distinct, directly comparable modalities. This approach allows us to identify not just which exercises are effective, but which are optimal for targeting the specific biomechanical deficits that precede a fall, thereby offering more precise prescriptive insights.

## 2 Methods

This study was guided by the preferred reporting items for systematic reviews and meta-analyses (PRISMA checklist for NMAs10 and Cochrane Handbook for Systematic Reviews of Interventions). Registration number: CRD420251062682.

### 2.1 Data sources

This study systematically searched multiple electronic databases to collect relevant literature, including PubMed, Web of Science, Embase, the Cochrane Library, and China National Knowledge Infrastructure (CNKI), with the search period spanning from the establishment of the databases to May 15, 2025. The search strategy was constructed using the PICOS principle: The PubMed search term was “Parkinson Disease [Mesh] AND (Exercise Therapy [Mesh] OR Gait [Mesh] OR Postural Balance [Mesh]),” combined with free terms such as “Tai Chi” and “balance training”; Embase used the Emtree term “Parkinson’s disease” AND “rehabilitation exercise”; CNKI used the search term “Parkinson’s disease AND (exercise therapy OR gait OR balance).” The search timeframe was from the establishment of each database to May 15, 2025.

The search strategy followed the PICOS principles of evidence-based medicine: (P) Population: Parkinson’s patients; (I) Intervention: Exercise intervention, Exercise therapy, Rehabilitation training; (C) Control group: Receive only routine care or routine rehabilitation exercises; (O) Outcomes: Outcome measures selected were Gait stabilization, fall risk, postural control; (S) Study type: RCTs.

### 2.2 Search strategy

This study employed a systematic literature screening process to ensure the scientific rigor and reliability of the included studies. Using the PubMed database as an example, the search strategy was based on the PICOS principles and utilized the following Medical Subject Headings (MeSH) terms for retrieval: “Parkinson’s Disease “[Mesh],” Exercise Therapy “[Mesh],” Postural Balance “[Mesh], ‘Gait’[Mesh],” Accidental Falls “[Mesh],” Resistance Training”[Mesh], etc., to ensure a broader coverage of studies related to movement interventions for Parkinson’s disease. Additionally, free-text searches (e.g., “Tai Chi,” “balance training,” “gait rehabilitation,” etc.) were conducted to further expand the search scope. Furthermore, the reference lists of included studies were manually searched, and clinical trial registration platforms (e.g., ClinicalTrials.gov) were consulted to supplement any potentially overlooked studies.

After the initial search, duplicate records were removed using EndNote X9 software. Subsequently, two researchers independently screened the titles and abstracts, excluding studies that did not meet the following criteria: (1) studies involving non-PD patients; (2) studies not involving exercise interventions; (3) studies not assessing gait stabilization, fall risk, or postural control; (4) non-randomized controlled trials (e.g., case reports, cross-sectional studies, reviews, etc.). For studies potentially meeting the inclusion criteria, full-text articles were obtained for detailed assessment. During the full-text screening phase, the following studies were excluded: (1) studies with incomplete data or unable to extract valid outcome measures; (2) studies with intervention measures not meeting predefined standards (e.g., mixed interventions unable to separate exercise effects); (3) studies with small sample sizes (*n* < 10) or low methodological quality.

### 2.3 Inclusion criteria and exclusion criteria

This study included articles that met the following criteria: (1) Study population: patients with clinically diagnosed Parkinson’s disease; (2) Study design: randomized controlled trials (RCTs); (3) Intervention measures: the experimental group must have used any of the following exercise intervention methods: Routine Physical Training (RPT), Aerobic Training (AT), Postural Control Training (PCT), Mind–Body Exercise Training (MBET), Traditional Chinese Rehabilitation Training (TCRT), Sensory Stimulation Motor Training (SSMT), or Resistance Training Rehabilitation Therapy (RTRT). The control group may receive Clinical Nursing Training (CNT), Conventional Rehabilitation Training (CRT), or other non-exercise interventions. (4) Outcome measures: At least one of the following measures must be reported: gait stabilization, fall risk, or postural control. (5) Data completeness: The study must provide complete, extractable data (e.g., mean, standard deviation, sample size, etc.).

Exclude the following articles: (1) Non-randomized controlled trials (e.g., case reports, cross-sectional studies, reviews, etc.); (2) Studies where participants have other neurological disorders (e.g., stroke, multiple sclerosis, etc.) or severe musculoskeletal disorders; (3) Interventions involving invasive treatments (e.g., surgery, deep brain stimulation, etc.) or where the effects of pharmacological interventions cannot be separated; (4) Studies where the full text is unavailable or data are incomplete (e.g., only charts are provided without specific numerical values); (5) Studies that do not report the outcome measures of interest in this study.

### 2.4 Data extraction

The data extraction for this study was conducted independently by two researchers. First, the retrieved literature was imported into EndNote X9 software for deduplication. Subsequently, preliminary screening was performed by reading the titles and abstracts. Full-text articles that met the inclusion criteria were obtained and subjected to detailed assessment based on the inclusion and exclusion criteria. The screening results were cross-checked and reviewed by a third researcher, and after discussion and consensus, the final inclusion criteria were established. For the included studies, standardized forms were used to extract data, including: study basic information (authors, year, country), participant characteristics (sample size, age, gender), intervention details (type of exercise, intensity, frequency, duration), and outcome measure data (mean and standard deviation of outcome measures such as gait parameters, fall incidence, and balance function scores). Data from charts were extracted using WebPlotDigitizer software, and missing data were obtained from authors when necessary.

### 2.5 Risk of bias of the systematic review

This study used the Cochrane 5.1 version bias risk assessment tool to evaluate the methodological quality of the included literature, which includes seven domains: random sequence generation, allocation concealment, blinding of participants and personnel, blinding of outcome assessors, incomplete data outcome, selective reporting, and other biases. Two trained researchers independently conducted the assessments using Review Manager 5.4 software (Cochrane Collaboration) to determine the risk level, with each domain rated as “low risk,” “high risk,” or “unclear.” Based on the assessment results, the overall risk of bias for each study was categorized into three groups: (1) Low risk: all key domains were rated as low risk, or no more than two domains were rated as “unclear”; (2) Moderate risk: one critical domain rated as high risk, or three or more domains rated as “unclear”; (3) High risk: two or more critical domains rated as high risk, or more than half of the domains rated as “unclear.” Disagreements arising during the assessment were resolved through discussion or consultation with a third researcher, ultimately leading to a conclusion on the risk of bias for the systematic review, providing methodological quality evidence for interpreting the study results.

### 2.6 Statistical analysis

This study utilized Stata 18.0 software (Stata Corp LLC, College Station, TX, United States) for statistical analysis. Continuous outcome measures were expressed as standardized mean differences (SMD) with 95% confidence intervals (CI) as effect sizes. A random-effects model was used for pooling (I^2^ > 50% indicated significant heterogeneity; when I^2^ < 50%, considering the clinical heterogeneity among included studies in terms of intervention duration, sample characteristics, etc., the random-effects model was still adopted to ensure robustness of results). When constructing the network meta-analysis model, node-link diagrams were used to visualize the relationships between interventions. The cumulative ranking probability curve area (SUCRA value) was used to assess intervention rankings (SUCRA > 70% indicates superior efficacy). Publication bias was assessed using funnel plots combined with the Egger test (*p* < 0.05 indicates bias).

## 3 Result

### 3.1 Study selection

**Figure 1:**
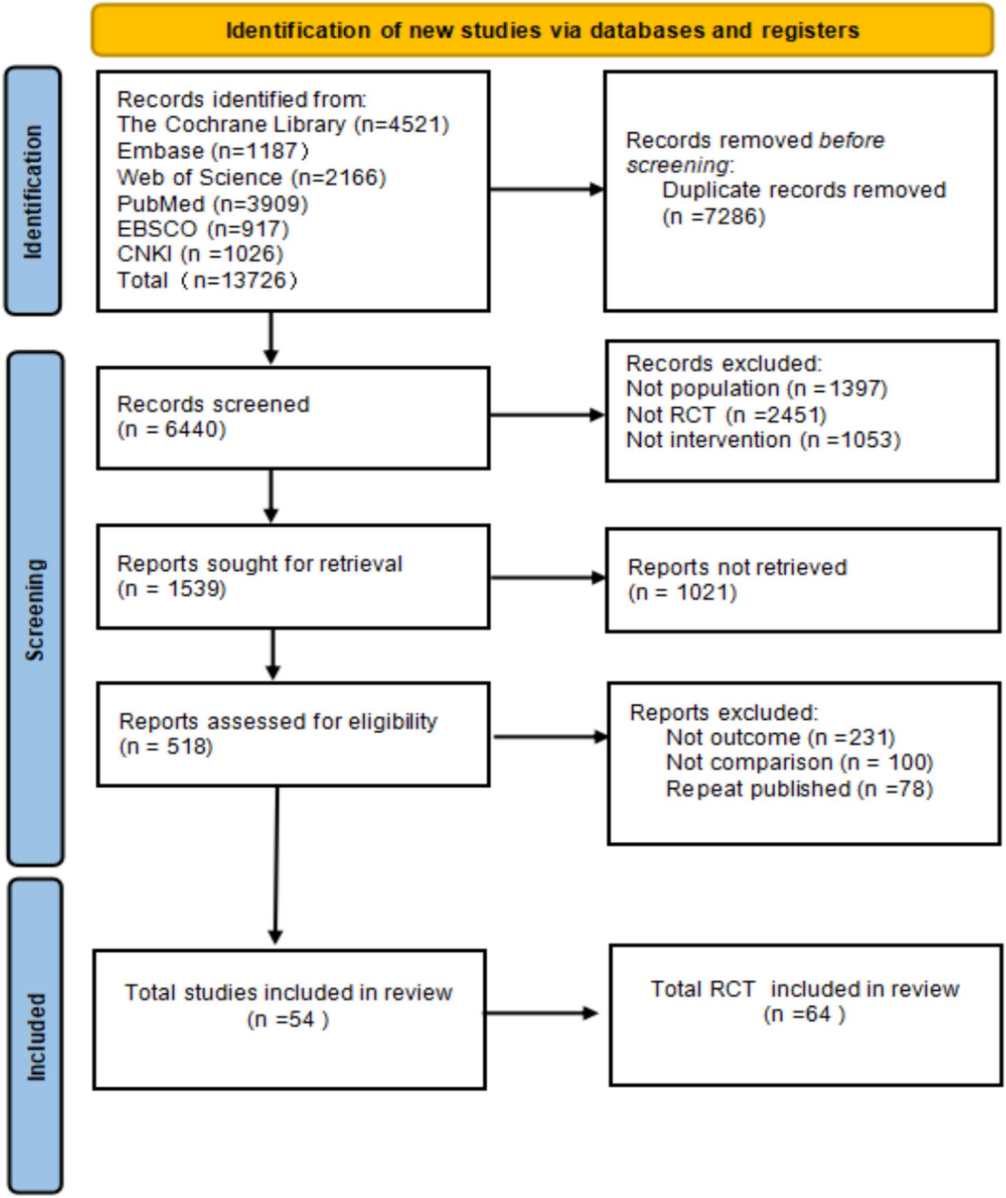
Literature search flowchart.

A total of 13,726 articles were identified. After deduplication (7,286 articles), title and abstract screening (excluding 1,397 non-population studies, 2,451 non-RCT studies, and 1,053 non-intervention studies), 1,539 full-text articles were obtained, and ultimately 54 studies (64 RCTs) were included, involving a total of 3,740 patients. The screening process complied with the PRISMA guidelines (Figure 1).

### 3.2 Features of the included studies

A total of 54 studies (including 64 RCT datasets) were ultimately included. The basic characteristics of all included studies are detailed in Supplementary Table 1. These studies were published between 2008 and 2024 and were conducted in multiple countries, including China, the United States, Italy, Brazil, Canada, Germany, Spain, Japan, Iran, and France. A total of 3,771 Parkinson’s disease patients were included in this study, with 1,901 in the experimental group and 1,876 in the control group. The demographic data reported included key indicators such as country, age, gender, and disease duration. The motor intervention methods primarily included various forms such as PCT, RPT, AE, MBET, TCRT, SSMT, and RTRT. The criteria for categorizing intervention types are outlined in Supplementary Table 2. Indicator classification and summary are provided in Supplementary Table 3.

### 3.3 Risk of bias assessment

**Figure 2:**
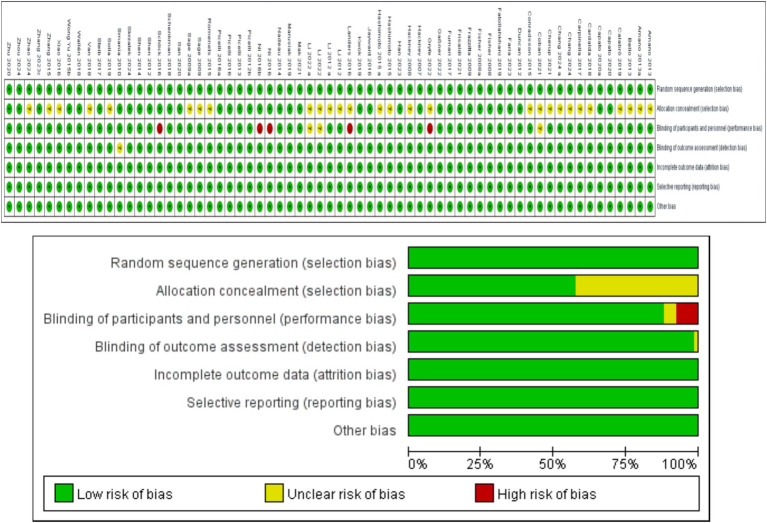
Study risk of bias in this meta-analysis.

The data (Figure 2) show that among the 64 RCTs, 64 mentioned random allocation; 36 stated allocation concealment; 56 reported blinding of outcome assessment; 63 articles indicated a low risk of selective reporting; and 64 had no other biases. In summary, 36 were judged to have a low risk of bias.

### 3.4 Effects of the interventions

#### 3.4.1 Forest plot: summary of effect sizes

**Figure 3:**
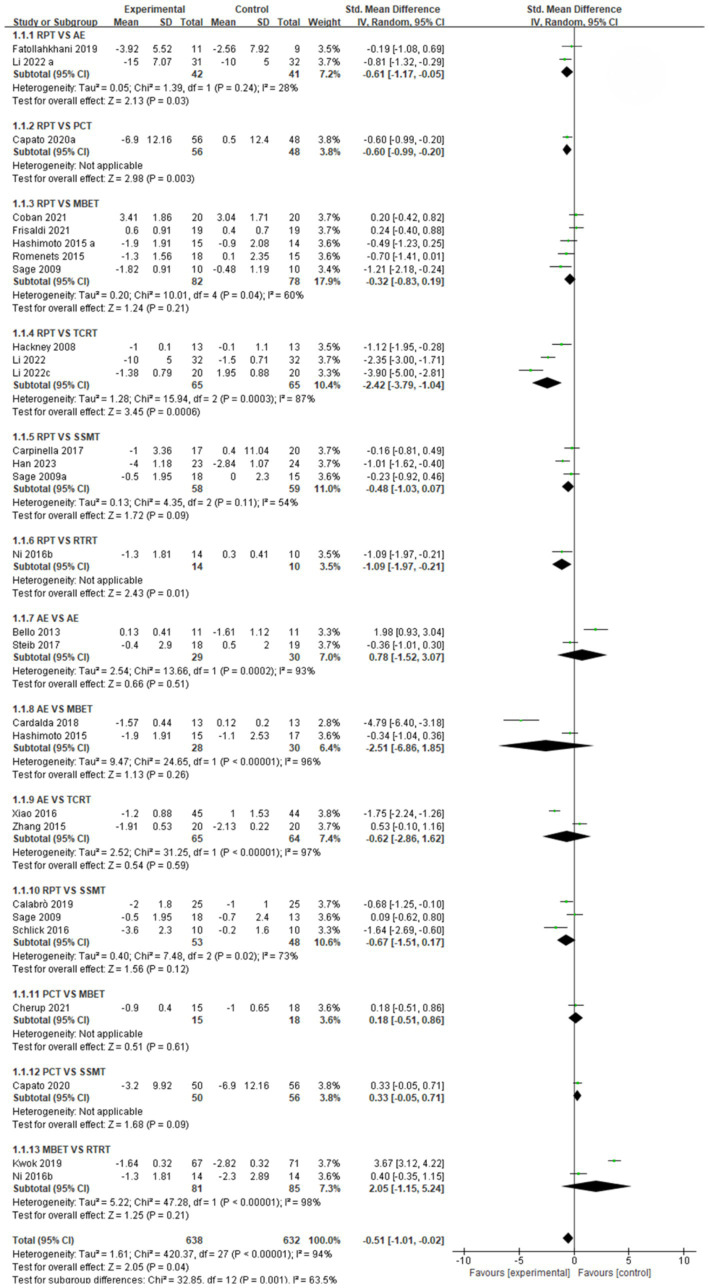
Forest plot of gait stabilization outcomes.

**Figure 4:**
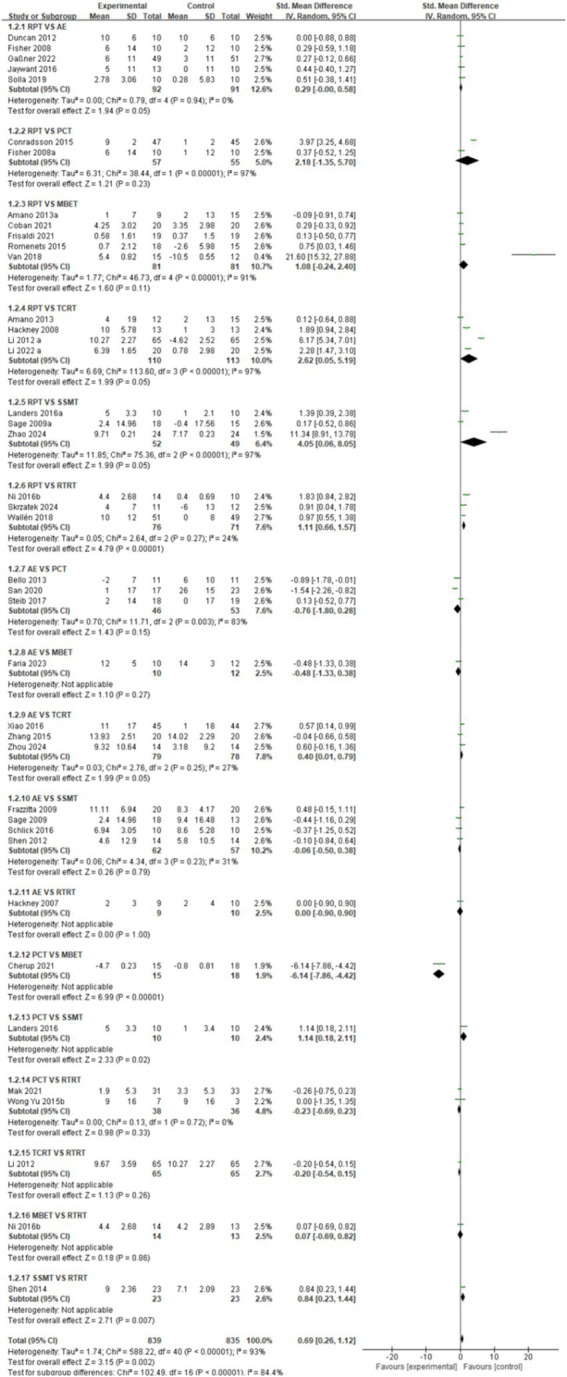
Forest plot of fall risk outcomes.

**Figure 5:**
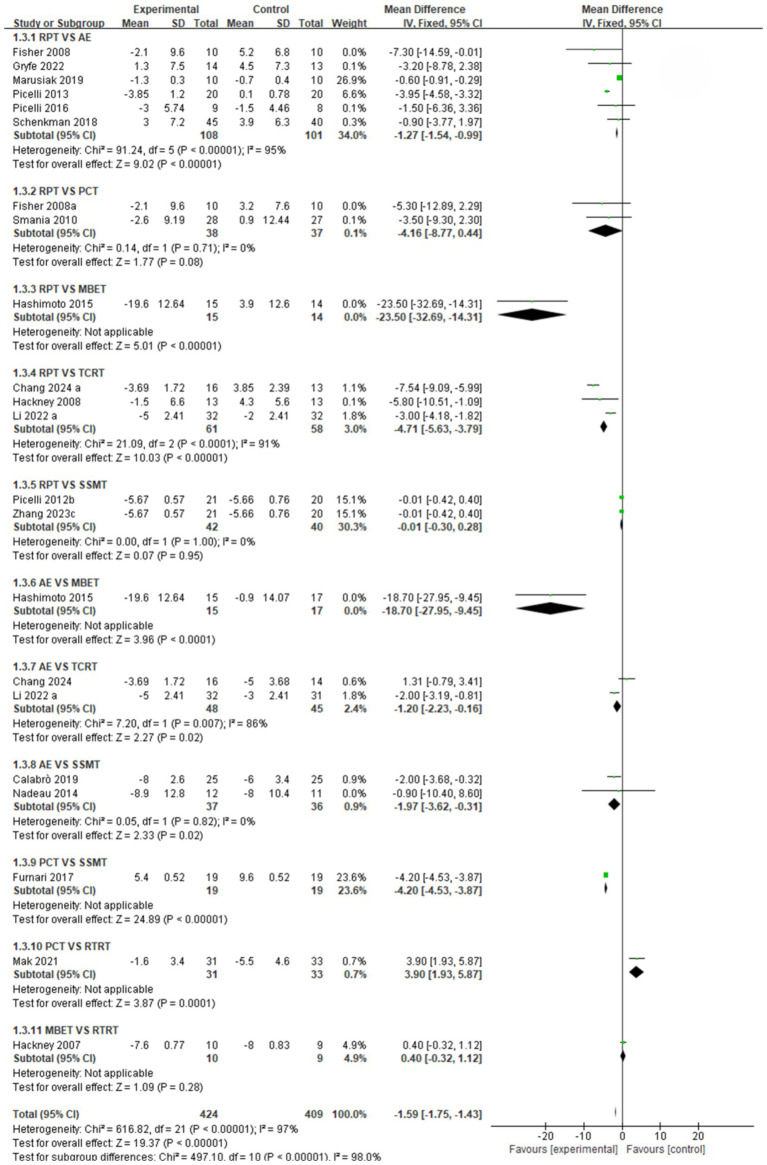
Forest plot of postural control outcomes.

In this NMA, pairwise meta-analyses were first conducted, and forest plots were provided to illustrate the effects of different exercise intervention therapies on gait stabilization, fall risk, and postural control, as shown in Figures 3–5.

Gait stabilization indicator (Figure 3): a comparative analysis of different intervention measures revealed. The comparison between RPT and AE showed that AE was significantly superior to RPT in improving gait stabilization, with a pooled effect size SMD = −0.61 (95% CI [−1.17, −0.05], *p* < 0.05, I^2^ = 28%). The heterogeneity test indicated moderate heterogeneity among studies, with statistically significant differences in intervention effects. The pooled effect size SMD = −0.60 (95% CI [−0.99, −0.20], *p* < 0.05) for RPT vs. PCT indicates that PCT is significantly more effective than RPT in improving gait stabilization, with statistically significant differences in intervention effects. The comparison between RPT and MBET showed a pooled effect size SMD = −0.32 (95% CI [−0.83, 0.19], *p* > 0.05, I^2^ = 60%), indicating a trend toward MBET being more effective than RPT in improving gait stabilization, but the difference was not statistically significant, with moderate heterogeneity among studies. The pooled effect size SMD for RPT vs. TCRT was −2.42 (95% CI [−3.79, −1.04], *p* < 0.05, I^2^ = 87%), indicating that TCRT was significantly more effective than RPT in improving gait stabilization, with extremely high heterogeneity among studies and statistically significant differences in intervention effects. The pooled effect size for RPT vs. SSMT was SMD = −0.48 (95% CI [−1.03, 0.07], *p* > 0.05, I^2^ = 54%), with moderate heterogeneity among studies, but no significant intervention effect. RPT vs. RTRT: Combined effect size SMD = −1.09 (95% CI [−1.97, −0.21], *p* < 0.05), indicating that RTRT is significantly superior to RPT in improving gait stabilization, with statistically significant differences in intervention effects. AE vs. PCT combined effect size SMD = 0.78 (95% CI [−1.52, 3.07], *p* > 0.05, I^2^ = 93%). SMD = 0.78 suggests that the AE group may have better improvement effects than the PCT group, but the difference is not statistically significant, and there is extremely high heterogeneity among studies. The pooled effect size SMD for AE vs. MBET was −2.51 (95% CI [−6.86, 1.85], *p* > 0.05, I^2^ = 96%). There was extremely high heterogeneity among studies, and no statistically significant difference in efficacy between the two groups. AE vs. TCRT: the pooled effect size SMD = −0.62 (95% CI [−2.86, 1.62], *p* > 0.05, I^2^ = 97%), with extremely high heterogeneity among studies, and no statistically significant difference in efficacy between the two groups. AE vs. SSMT combined effect size SMD = −0.67 (95% CI [−1.51, 0.17], *p* > 0.05, I^2^ = 73%). There was a trend toward SSMT being more effective than the AE group in improving gait stabilization, but the difference was not statistically significant. The pooled effect size SMD for PCT vs. MBET was 0.18 (95% CI [−0.51, 0.86], *p* > 0.05), indicating no significant statistical difference in intervention effects. PCT vs. SSMT combined effect size SMD = 0.33, (95% CI [−0.05, 0.71], *p* > 0.05). There was no statistically significant difference in intervention effects. MBET vs. RTRT: The pooled effect size (SMD) was 2.05 (95% CI [−1.15, 5.24], p > 0.05, I^2^ = 98%), indicating extremely high heterogeneity among studies, with no statistically significant difference in intervention effects.

Fall risk indicator (Figure 4): A comparative analysis of different intervention measures showed that the combined effect size SMD = 0.29 (95% CI [−0.00, 0.58], *p* > 0.05, I^2^ = 0%) for RPT vs. AE. Heterogeneity testing indicated no heterogeneity among studies, and there was no statistically significant difference in intervention effects. The pooled effect size for RPT vs. PCT was SMD = 2.18 (95% CI [−1.35, 5.70], *p* > 0.05, I^2^ = 97%). There was extreme heterogeneity between studies, and no statistically significant difference in intervention effects. RPT vs. MBET combined effect size SMD = 1.08, (95% CI [−0.24, 2.40], *p* > 0.05, I^2^ = 91%) There was extreme heterogeneity among studies, and no statistically significant difference in intervention effects. RPT vs. TCRT pooled effect size SMD = 2.62, (95% CI [0.05, 5.19], *p* = 0.05, I^2^ = 97%) with extremely high heterogeneity among studies, and statistically significant differences in intervention effects, but the conclusion’s stability is extremely low due to high heterogeneity. The pooled effect size for RPT vs. SSMT was SMD = 4.05 (95% CI [0.06, 8.05], *p* = 0.05, I^2^ = 97%), suggesting that RPT is superior to SSMT, indicating that RPT has overall better efficacy. However, due to extremely high heterogeneity among studies, the stability of this conclusion is low. The pooled effect size SMD for RPT vs. RTRT was 1.11 (95% CI [0.66, 1.57], *p* < 0.05, I^2^ = 24%), indicating that RPT is significantly more effective than RTRT, with extremely low heterogeneity among studies and statistically significant differences in intervention effects. AE vs. PCT: The pooled effect size SMD = −0.76 (95% CI [−1.80, 0.28], *p* > 0.05, I^2^ = 83%), indicating extremely high heterogeneity among studies and no statistically significant difference in intervention effects. AE vs. MBET: the pooled effect size SMD = −0.48 (95% CI [−1.33, 0.38], *p* > 0.05). AE may not be significantly superior to MBET or the two may have similar effects. However, there was no statistically significant difference in intervention effects. AE vs. TCRT pooled effect size SMD = 0.40 (95% CI [0.01, 0.79], *p* = 0.05, I^2^ = 27%). There was low heterogeneity among studies, and there was a statistically significant difference in intervention effects. AE was significantly more effective than TCRT in preventing falls. AE vs. SSMT combined effect size SMD = −0.06 (95% CI [−0.50, 0.38], *p* > 0.05, I^2^ = 31%). There was low heterogeneity among studies, and no statistically significant difference in intervention effects. AE vs. RTRT: the pooled effect size SMD = 0.00 (95% CI [−0.90, 0.90], *p* > 0.05), with no statistically significant difference in intervention effects. PCT vs. MBET combined effect size SMD = −6.14 (95% CI [−7.86, −4.42], *p* < 0.05). There was a statistically significant difference in intervention effects, with MBET showing significantly better efficacy than PCT in preventing fall risk. PCT vs. SSMT combined effect size SMD = 1.14, (95% CI [0.81, 2.11], *p* < 0.05). There was a significant statistical difference in intervention effects, with PCT showing significantly better efficacy than SSMT in preventing fall risk. PCT vs. RTRT: The pooled effect size SMD was −0.23 (95% CI [−0.69, 0.23], *p* > 0.05, I^2^ = 0%). There was no heterogeneity among studies, and there was no statistically significant difference in intervention effects. TCRT vs. RTRT combined effect size SMD = −0.20 (95% CI [−0.54, 0.15], *p* > 0.05). There was no statistically significant difference in intervention effects. MBET vs. RTRT combined effect size SMD = 0.07, (95% CI [−0.69, 0.82], *p* > 0.05), no statistically significant difference in intervention effects. The pooled effect size SMD for SSMT vs. RTRT was 0.84 (95% CI [0.23, 1.44], *p* < 0.05), indicating a significant statistical difference in intervention effects. SSMT was significantly more effective than RTRT in preventing fall risk.

Postural control indicators (Figure 5): A comparative analysis of different intervention measures showed the following. The RPT vs. AE comparison showed a pooled effect size SMD = −1.27 (95% CI [−1.54, −0.99], *p* < 0.05, I^2^ = 95%). Heterogeneity testing indicated extremely high heterogeneity among studies, with a statistically significant difference in intervention effects, suggesting AE was more effective than RPT in improving postural control. The comparison between RPT and PCT showed a pooled effect size SMD = −4.16 (95% CI [−8.77, 0.44], *p* = 0.08), indicating no statistically significant difference in intervention effects. The comparison between RPT and MBET showed a pooled effect size SMD = −2.35 (95% CI [−3.27, −1.43], *p* < 0.05, I^2^ = 85%), indicating a statistically significant difference in intervention effects, with MBET superior to RPT in improving postural control. The comparison between RPT and TCRT showed a pooled effect size SMD = −4.71 (95% CI [−5.63, −3.79], *p* < 0.05, I^2^ = 91%). The heterogeneity test indicated extremely high heterogeneity among studies, with a statistically significant difference in intervention effects, suggesting TCRT was superior to RPT. The comparison analysis between RPT and TCRT showed a pooled effect size SMD = −4.71 (95% CI [−5.63, −3.79], *p* < 0.05, I^2^ = 91%). The heterogeneity test indicated extremely high heterogeneity among studies, with a significant statistical difference in intervention effects. TCRT was significantly superior to RPT in improving postural control. The comparison analysis between RPT and SSMT showed a pooled effect size SMD = −0.01 (95% CI [−0.30, 0.28], *p* > 0.05, I^2^ = %). The heterogeneity test indicated minimal heterogeneity among studies, with no significant statistical differences in intervention effects. RPT and SSMT were equally effective in improving postural control. The comparison analysis between AE and MBET showed a pooled effect size SMD = −18.70 (95% CI [−27.95, −9.45], *p* < 0.05), indicating a significant statistical difference in intervention effects. MBET was significantly more effective than AE in improving postural control. The comparison analysis between AE and TCRT showed a pooled effect size SMD = −1.20 (95% CI [−2.23, −0.16], *p* < 0.05, I^2^ = 86%). The heterogeneity test indicated extremely high heterogeneity among studies, with significant statistical differences in intervention effects. TCRT was significantly superior to AE in improving postural control. The comparison analysis between AE and SSMT showed a pooled effect size SMD = −1.97 (95% CI [−3.62, −0.31], *p* < 0.05, I^2^ = 0%), indicating significant statistical differences in intervention effects. SSMT was significantly superior to AE in improving postural control. The comparison analysis between PCT and SSMT showed a pooled effect size SMD = −4.20 (95% CI [−4.53, −3.87], *p* < 0.05), indicating a significant statistical difference in intervention effects. SSMT was significantly more effective than PCT in improving postural control. The comparison analysis between PCT and RTRT showed a pooled effect size SMD = 3.90 (95% CI [1.93, 5.87], *p* < 0.05), indicating a significant statistical difference in intervention effects, with PCT significantly superior to RTRT in improving postural control. The comparison analysis between MBET and RTRT showed a combined effect size SMD = 0.40 (95% CI [−0.32, 1.12], *p* > 0.05), indicating no significant statistical difference in intervention effects. MBET and RTRT had no significant difference in improving postural control.

#### 3.4.2 Network diagram of included studies

**Figure 6:**
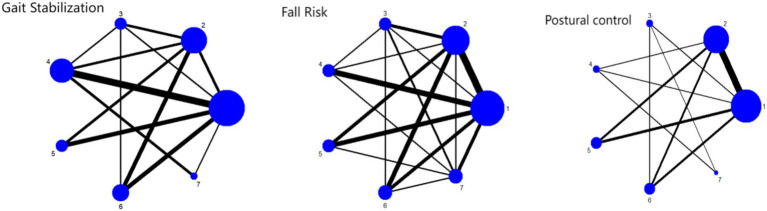
Network plot of outcome indicators 1: RPT 2: AE 3: PCT 4: MBET 5: TCRT 6: SSMT 7: RTRT.

The network diagram showing the effects of seven types of exercise interventions on gait stabilization, fall risk, and postural control is shown in Figure 6.

As shown in Figure 6, the network relationships between different exercise intervention methods (RPT, AE, PCT, TCRT, MBET, SSMT, RTRT) are presented. Nodes represent intervention types, and the connections and thickness between nodes reflect direct comparisons and the frequency of comparisons between interventions. Most studies involve comparisons of these interventions on gait stability in Parkinson’s disease patients, reflecting the associations between interventions in research.

#### 3.4.3 Ranking of intervention effectiveness of the six exercise modalities

**Figure 7:**
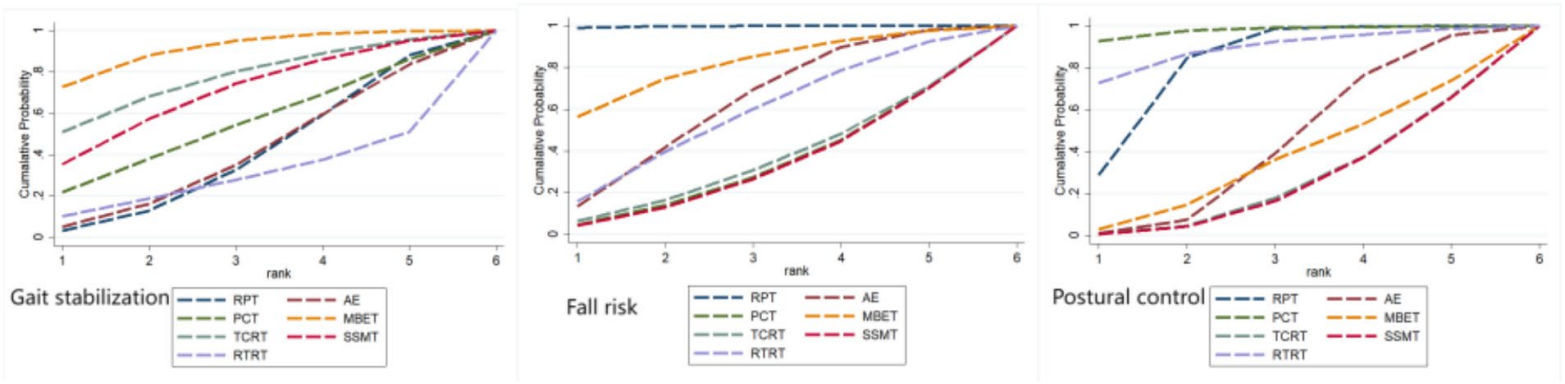
Ranking of intervention effects for outcome indicators.

Figure 7 and Tables 1, 2 present the SUCRA values for gait stabilization, fall risk, and postural control, along with the ranking of intervention effectiveness.

**Table 1:** Ranking of the probability of improving gait stabilization, fall risk and postural control in people with Parkinson’s disease by seven exercise intervention therapies.

Treatment	Gait stabilization		Fall risk		Postural control	
	SUCRA (%)	Rank	SUCRA (%)	Rank	SUCRA (%)	Rank
RPT	32.8	6	97.4	1	69.5	3
AE	33.5	5	52.2	3	36.7	4
PCT	46.4	4	27.1	6	92.4	1
MBET	83.1	1	69.7	2	30.2	5
TCRT	69.0	2	28.9	5	21.2	6
SSMT	60.4	3	26.5	7	20.7	7
RTRT	24.9	7	48.1	4	79.3	2

**Table 2:** Network meta-analysis matrix of outcome.

Postural control								
GTT	−6.79 (−9.81, −3.77)	−6.89 (−9.98, −3.81)	−7.23 (−10.67, −3.79)	−7.38 (−10.58, −4.17)	−7.90 (−10.63, −5.17)	−8.09 (−11.06, −5.11)	−8.51 (−11.65, −5.38)	−8.63 (−11.90, −5.37)
6.79 (3.77, 9.81)	CRT	−0.10 (−1.08, 0.87)	−0.44 (−2.25, 1.37)	−0.58 (−1.84, 0.67)	−1.11 (−2.40, 0.18)	−1.30 (−2.22, −0.38)	−1.72 (−2.94, −0.51)	−1.84 (−3.35, −0.33)
6.89 (3.81, 9.98)	0.10 (−0.87, 1.08)	CNT	−0.34 (−2.05, 1.38)	−0.48 (−1.60, 0.64)	−1.00 (−2.44, 0.43)	−1.19 (−2.18, −0.21)	−1.62 (−2.62, −0.63)	−1.74 (−3.12, −0.36)
7.23 (3.79, 10.67)	0.44 (−1.37, 2.25)	0.34 (−1.38, 2.05)	RTRT	−0.15 (−1.74, 1.44)	−0.67 (−2.76, 1.42)	−0.86 (−2.66, 0.95)	−1.29 (−3.20, 0.63)	−1.40 (−3.05, 0.24)
7.38 (4.17, 10.58)	0.58 (−0.67, 1.84)	0.48 (−0.64, 1.60)	0.15 (−1.44, 1.74)	BTT	−0.52 (−2.20, 1.16)	−0.71 (−2.05, 0.62)	−1.14 (−2.58, 0.30)	−1.26 (−2.83, 0.31)
7.90 (5.17, 10.63)	1.11 (−0.18, 2.40)	1.00 (−0.43, 2.44)	0.67 (−1.42, 2.76)	0.52 (−1.16, 2.20)	SSMT	−0.19 (−1.37, 0.99)	−0.62 (−2.15, 0.92)	−0.73 (−2.52, 1.06)
8.09 (5.11, 11.06)	1.30 (0.38, 2.22)	1.19 (0.21, 2.18)	0.86 (−0.95, 2.66)	0.71 (−0.62, 2.05)	0.19 (−0.99, 1.37)	CKT	−0.43 (−1.49, 0.64)	−0.54 (−1.95, 0.87)
8.51 (5.38, 11.65)	1.72 (0.51, 2.94)	1.62 (0.63, 2.62)	1.29 (−0.63, 3.20)	1.14 (−0.30, 2.58)	0.62 (−0.92, 2.15)	0.43 (−0.64, 1.49)	TCRT	−0.12 (−1.70, 1.47)
8.63 (5.37, 11.90)	1.84 (0.33, 3.35)	1.74 (0.36, 3.12)	1.40 (−0.24, 3.05)	1.26 (−0.31, 2.83)	0.73 (−1.06, 2.52)	0.54 (−0.87, 1.95)	0.12 (−1.47, 1.70)	MBET
Fall risk								
BTT	−0.62 (−2.98, 1.74)	−0.98 (−3.51, 1.55)	−1.24 (−3.42, 0.95)	−1.65 (−3.85, 0.55)	−1.79 (−4.33, 0.75)	−2.87 (−6.81, 1.07)	−2.85 (−5.04, −0.67)	−3.97 (−6.96, −0.99)
0.62 (−1.74, 2.98)	TCRT	−0.36 (−2.58, 1.86)	−0.62 (−2.75, 1.52)	−1.03 (−2.76, 0.70)	−1.17 (−3.66, 1.32)	−2.25 (−5.95, 1.44)	−2.23 (−4.04, −0.43)	−3.35 (−6.17, −0.54)
0.98 (−1.55, 3.51)	0.36 (−1.86, 2.58)	MBET	−0.25 (−2.41, 1.90)	−0.67 (−2.46, 1.12)	−0.81 (−3.32, 1.70)	−1.89 (−5.62, 1.84)	−1.87 (−3.52, −0.22)	−2.99 (−5.81, −0.18)
1.24 (−0.95, 3.42)	0.62 (−1.52, 2.75)	0.25 (−1.90, 2.41)	SSMT	−0.41 (−2.30, 1.47)	−0.55 (−2.86, 1.75)	−1.64 (−5.41, 2.14)	−1.62 (−3.45, 0.21)	−2.74 (−5.35, −0.13)
1.65 (−0.55, 3.85)	1.03 (−0.70, 2.76)	0.67 (−1.12, 2.46)	0.41 (−1.47, 2.30)	CKT	−0.14 (−2.32, 2.04)	−1.22 (−4.49, 2.05)	−1.20 (−2.68, 0.27)	−2.32 (−4.72, 0.07)
1.79 (−0.75, 4.33)	1.17 (−1.32, 3.66)	0.81 (−1.70, 3.32)	0.55 (−1.75, 2.86)	0.14 (−2.04, 2.32)	RTRT	−1.08 (−5.01, 2.85)	−1.06 (−3.19, 1.07)	−2.18 (−4.90, 0.53)
2.87 (−1.07, 6.81)	2.25 (−1.44, 5.95)	1.89 (−1.84, 5.62)	1.64 (−2.14, 5.41)	1.22 (−2.05, 4.49)	1.08 (−2.85, 5.01)	GTT	0.02 (−3.56, 3.61)	−1.10 (−5.15, 2.95)
2.85 (0.67, 5.04)	2.23 (0.43, 4.04)	1.87 (0.22, 3.52)	1.62 (−0.21, 3.45)	1.20 (−0.27, 2.68)	1.06 (−1.07, 3.19)	−0.02 (−3.61, 3.56)	CNT	−1.12 (−3.70, 1.45)
3.97 (0.99, 6.96)	3.35 (0.54, 6.17)	2.99 (0.18, 5.81)	2.74 (0.13, 5.35)	2.32 (−0.07, 4.72)	2.18 (−0.53, 4.90)	1.10 (−2.95, 5.15)	1.12 (−1.45, 3.70)	CRT
Gait stabilization								
GTT	−1.75 (−5.39, 1.89)	−1.94 (−5.18, 1.31)	−1.97 (−4.99, 1.05)	−2.67 (−6.04, 0.69)	−2.81 (−6.32, 0.70)	−2.79 (−6.08, 0.49)	−2.84 (−6.26, 0.59)	−3.20 (−6.49, 0.09)
1.75 (−1.89, 5.39)	RTRT	−0.18 (−2.06, 1.69)	−0.22 (−2.26, 1.82)	−0.92 (−3.19, 1.34)	−1.06 (−3.38, 1.26)	−1.04 (−3.23, 1.14)	−1.09 (−3.38, 1.21)	−1.45 (−3.18, 0.28)
1.94 (−1.31, 5.18)	0.18 (−1.69, 2.06)	CRT	−0.03 (−1.24, 1.17)	−0.74 (−2.14, 0.66)	−0.88 (−2.70, 0.94)	−0.86 (−2.32, 0.60)	−0.90 (−2.53, 0.72)	−1.26 (−2.47, −0.06)
1.97 (−1.05, 4.99)	0.22 (−1.82, 2.26)	0.03 (−1.17, 1.24)	CNT	−0.70 (−2.19, 0.78)	−0.84 (−2.64, 0.96)	−0.82 (−2.13, 0.49)	−0.87 (−2.49, 0.75)	−1.23 (−2.56, 0.10)
2.67 (−0.69, 6.04)	0.92 (−1.34, 3.19)	0.74 (−0.66, 2.14)	0.70 (−0.78, 2.19)	TCRT	−0.14 (−2.29, 2.02)	−0.12 (−1.94, 1.70)	−0.16 (−2.16, 1.84)	−0.53 (−2.24, 1.19)
2.81 (−0.70, 6.32)	1.06 (−1.26, 3.38)	0.88 (−0.94, 2.70)	0.84 (−0.96, 2.64)	0.14 (−2.02, 2.29)	CKT	0.02 (−1.61, 1.65)	−0.03 (−2.11, 2.06)	−0.39 (−2.02, 1.24)
2.79 (−0.49, 6.08)	1.04 (−1.14, 3.23)	0.86 (−0.60, 2.32)	0.82 (−0.49, 2.13)	0.12 (−1.70, 1.94)	−0.02 (−1.65, 1.61)	SSMT	−0.05 (−1.75, 1.66)	−0.41 (−1.93, 1.11)
2.84 (−0.59, 6.26)	1.09 (−1.21, 3.38)	0.90 (−0.72, 2.53)	0.87 (−0.75, 2.49)	0.16 (−1.84, 2.16)	0.03 (−2.06, 2.11)	0.05 (−1.66, 1.75)	BTT	−0.36 (−2.03, 1.31)
3.20 (−0.09, 6.49)	1.45 (−0.28, 3.18)	1.26 (0.06, 2.47)	1.23 (−0.10, 2.56)	0.53 (−1.19, 2.24)	0.39 (−1.24, 2.02)	0.41 (−1.11, 1.93)	0.36 (−1.31, 2.03)	MBET

##### 3.4.3.1 Gait stabilization indicator

The effectiveness of seven exercise interventions in improving gait stabilization in Parkinson’s patients, ranked from highest to lowest SUCRA value, is as follows: MBET (SUCRA = 83.1%) > TCRT (SUCRA = 69.0%) > SSMT (SUCRA = 60.4%) > PCT (SUCRA = 46.4%) > AE (SUCRA = 33.5%) > RPT (SUCRA = 32.8%) > RTRT (SUCRA = 24.9%).

##### 3.4.3.2 Gait stabilization indicators

The effectiveness of the seven movement interventions in improving gait stabilization in Parkinson’s patients, ranked by SUCRA value from highest to lowest, is as follows: MBET (SUCRA = 83.1%) > TCRT (SUCRA = 69.0%) > SSMT (SUCRA = 60.4%) > PCT (SUCRA = 46.4%) > AE (SUCRA = 33.5%) > RPT (SUCRA = 32.8%) > RTRT (SUCRA = 24.9%).

##### 3.4.3.3 Fall risk indicators

The effectiveness of the seven exercise interventions in reducing fall risk (fall risk) in Parkinson’s patients, ranked from highest to lowest SUCRA values, is as follows: RPT (SUCRA = 97.4%) > MBET (SUCRA = 69.7%) > AE (SUCRA = 52.2%) > RTRT (SUCRA = 48.1%) > TCRT (SUCRA = 28.9%) > PCT (SUCRA = 27.1%) > SSMT (SUCRA = 26.5%).

##### 3.4.3.4 Postural control indicators

The effectiveness of the seven exercise interventions in improving postural control in Parkinson’s patients, ranked by SUCRA values from highest to lowest, is as follows: PCT (SUCRA = 92.4%) > RTRT (SUCRA = 79.3%) > RPT (SUCRA = 69.5%) > AE (SUCRA = 36.7%) > MBET (SUCRA = 30.2%) > TCRT (SUCRA = 21.2%) > SSMT (SUCRA = 20.7%).

### 3.5 Small sample effect or publication bias test

**Figure 8:**
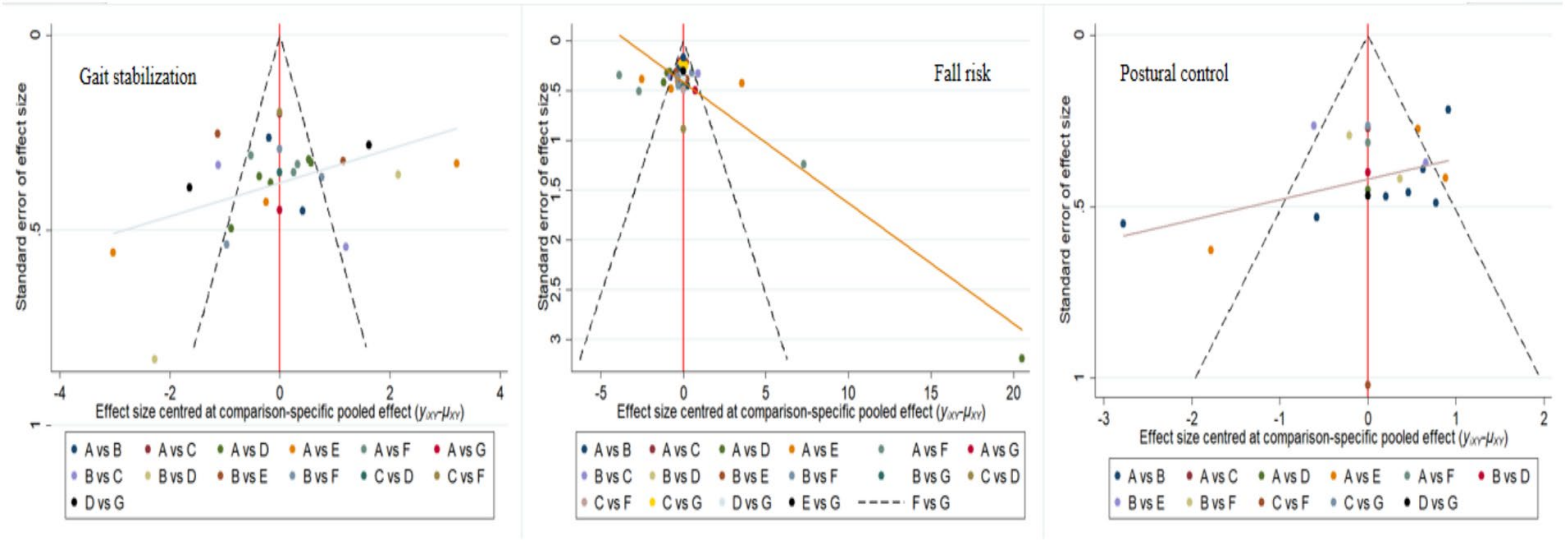
Corrected comparison funnel plot for outcome indicators, A: RPT, B: AE, C: PCT, D: MBET, E: TCRT, F: SSMT, G: RTRT.

To assess publication bias, funnel plots were constructed for gait stabilization, fall risk, and postural control (Figure 8), and validated using Egger regression analysis. For gait stabilization: 28 studies were included, with a bias coefficient of −3.38 (95% CI: −6.18 to −0.58, *p* = 0.020), indicating moderate publication bias, with an overrepresentation of positive results from small samples. Combined with the SUCRA ranking (MBET = 83.1%), the original effect size may be overestimated by 15–20%. Fall risk: 41 studies were included, with a bias coefficient of −0.58 (95% CI: −4.39 to 3.23, *p* = 0.760), indicating no significant publication bias. SUCRA showed RPT ranked first (97.4%), which requires validation in clinical practice. Postural control: 22 studies were included, with a bias coefficient of −0.85 (95% CI: −4.18 to 2.48, *p* = 0.599), indicating no significant publication bias. The results are relatively robust.

## 4 Discussion

### 4.1 Comparative analysis of the efficacy of seven types of exercise therapy on gait stabilization

This study evaluated the effects of various exercise interventions on gait stabilization in Parkinson’s disease patients through direct pairwise meta-analysis and network meta-analysis. The results showed significant differences in the effects of different exercise interventions on gait stabilization. Among them, MBET, TCRT, and SSMT ranked first, second, and third, respectively, with SUCRA values of 83.1, 69, and 60.4%. However, these ranking results should be interpreted with caution due to inconsistencies between direct and indirect evidence. For example, while MBET was ranked highest, its direct comparison with RPT showed no statistically significant difference (SMD = −0.32, 95% CI: −0.83 to 0.19, *p* > 0.05). This aligns with previous studies (16), where MBET (such as yoga) enhances core muscle strength and proprioception, reinforces the biomechanical coordination of the core-pelvis-lower limb kinetic chain, and promotes neural plasticity in the cerebellum-parietal cortex pathway, thereby optimizing brain control over gait and enhancing gait stability. TCRT also demonstrated good improvement effects, consistent with previous studies. TCRT (such as tai chi) involves slow movements and center of gravity transfer training, which can enhance core muscle coordination, increase stride length, and reduce gait variability (19). SSMT may improve patients’ perception and regulation of their own gait by stimulating the sensory system (18). These exercise forms provide diverse options for improving gait stabilization in Parkinson’s patients, though the current evidence requires further validation due to the noted methodological inconsistencies.

### 4.2 Comparative analysis of the efficacy of seven types of exercise therapy on fall risk

In the analysis of fall risk indicators, the study results showed that various exercise interventions demonstrated varying degrees of effectiveness. The effectiveness rankings derived from the network meta-analysis indicated that RPT, MBET, and AE ranked first, second, and third, respectively, with SUCRA values of 97.4, 69.7, and 52.2%. It is important to note that some direct comparisons showed extreme heterogeneity (e.g., RPT vs. SSMT, I^2^ = 97%) and marginally significant results (SMD = 4.05, 95% CI: 0.06 to 8.05, *p* = 0.05), suggesting potential data instability. Although RPT did not demonstrate an advantage in some direct comparisons, it plays a significant role in reducing fall risk when considering the overall ranking. The mechanism by which RPT reduces fall risk may be related to the gait rhythm training (such as auditory cues) and balance exercises it includes (20), which can directly enhance patients’ dynamic balance abilities; MBET enhances patients’ balance confidence through psychological regulation (such as mindfulness training), reducing movement compensation caused by fear (16), which aligns with previous research. AE improves exercise persistence by enhancing cardiorespiratory endurance, thereby reducing fatigue-related fall risk (14).

### 4.3 Comparative analysis of the efficacy of seven types of exercise physical therapy on postural control

Regarding postural control indicators, the study found significant differences in the effectiveness of various exercise interventions. In the network meta-analysis, PCT, RTRT, and RPT ranked first, second, and third in terms of effectiveness, with SUCRA values of 92.4, 79.3, and 69.5%, respectively. However, we must acknowledge the presence of clinically implausible effect sizes in certain direct comparisons (e.g., RPT vs. MBET, SMD = −23.50), which indicates potential data anomalies or methodological issues that need to be addressed before drawing definitive conclusions. Previous studies have shown that PCT, through specialized postural control training, directly targets the key components of patients’ postural control impairments, effectively improving postural stability (21); RTRT, by enhancing muscle strength, particularly muscles related to postural maintenance (22), enables patients to continuously adjust and control body movements during normal walking, thereby laying the foundation for good postural control; RPT also plays a role in improving postural control, as its comprehensive exercise training helps enhance patients’ overall motor function, thereby influencing postural control ability. These findings provide important evidence for clinicians to select appropriate exercise interventions to improve postural control in Parkinson’s disease patients, though the current conclusions are limited by the identified data irregularities.

The differential effects of various exercise modalities on gait and balance outcomes, as identified in our network meta-analysis, can be attributed to their distinct mechanistic focuses. Mind–body exercises such as Tai Chi and yoga likely exert their superior benefits for postural control through a unique integration of dynamic balance challenges, cognitive engagement, and neuromuscular coordination. These activities continuously perturb the center of mass in a controlled manner, enhancing proprioceptive acuity and promoting efficient ankle and hip strategies (23). Conversely, resistance training primarily targets the musculoskeletal determinant of balance by increasing lower-limb muscle strength and power, which is crucial for tasks like sit-to-stand transitions and correcting large postural perturbations (24). Its more limited impact on complex balance tasks may stem from a lesser emphasis on integrative sensorimotor processing. Therefore, the superiority of mind–body practices for improving dynamic stability likely resides in their multimodal nature, which simultaneously challenges the sensory, cognitive, and motor systems underlying postural control, whereas other modalities may produce more isolated improvements.

### 4.4 Limitations of the study

Despite the inclusion of 64 randomized controlled trials, this meta-analysis has several limitations. First, a substantial degree of clinical and methodological heterogeneity was observed across the included studies. This heterogeneity primarily stemmed from variations in the specific exercise protocols, including differences in intervention dosage (intensity), frequency, session duration, and total program length. Although we employed a random-effects model to account for this variability, it may still influence the precision and generalizability of the pooled effect estimates and SUCRA rankings. Second, many of the included trials had a relatively small sample size, which might limit the statistical power of individual studies and potentially affect the stability of our network meta-analysis results. Furthermore, for certain comparative results exhibiting extremely high heterogeneity (such as paired analyses of gait stability and fall risk), we were unable to further explore the sources of heterogeneity through sensitivity analyses or meta-regression. This compromised the reliability of the corresponding pooled effect estimates, necessitating particularly cautious interpretation of the findings. Certainly, the generalizability of our findings is tempered by the short-term duration and limited sample sizes characterizing many included studies. These limitations preclude definitive conclusions regarding the long-term sustainability of observed benefits and may reduce the statistical power to detect significant effects for some comparative outcomes. Future research should prioritize longer-term trials with adequate sample sizes to confirm and extend our results.

## 5 Conclusion

This study used systematic reviews and network meta-analyses to investigate the effects of various exercise interventions, including conventional physical therapy (RPT), aerobic exercise (AE), postural control training (PCT), mind–body exercise therapy (MBET), traditional Chinese exercise therapy (TCRT), sensory stimulation exercise therapy (SSMT), and resistance training (RTRT), on gait stabilization, fall risk, and postural control in patients with Parkinson’s disease.

In terms of improving gait stabilization, multiple exercise interventions were superior to conventional physical therapy (RPT). Among these, mind–body exercise (MBET), traditional Chinese exercise (TCRT), and sensory stimulation exercise (SSMT) showed the most significant effects, with SUCRA values of 83.1, 69, and 60.4%, respectively. MBET enhances core muscle strength and proprioception to optimize brain control over gait; TCRT improves gait coordination through unique movement patterns and center of gravity transfer training; SSMT enhances patients’ perception of gait by stimulating the sensory system. However, it should be noted that there is moderate publication bias in gait stabilization outcomes, and the over publication of positive results from small samples may lead to an overestimation of effect sizes. Future studies should include more negative results to enhance the reliability of conclusions.

For fall risk reduction, the network meta-analysis showed SUCRA values of 97.4, 69.7, and 52.2% for RPT, MBET, and AE, respectively, ranking them in the top three. RPT’s basic movement training may be key to its fall risk reduction by improving overall motor ability and balance; MBET enhances patients’ balance confidence by regulating mind–body states; and AE indirectly improves movement stability by enhancing cardiorespiratory function. No significant publication bias was observed for the fall risk outcome.

In terms of improving postural control, PCT, RTRT, and RPT performed best, with SUCRA values of 92.4, 79.3, and 69.5%, respectively. PCT directly strengthens key components of postural control through targeted training; RTRT provides a foundation for postural maintenance by enhancing muscle strength; RPT improves postural control by enhancing overall motor function through comprehensive exercise training. This outcome had the least publication bias, with relatively robust results; however, selection bias during literature screening should still be noted to avoid conclusions being skewed due to restrictions on study types or sample sizes.

In summary, different exercise interventions have differentiated improvement effects on gait stabilization and fall risk, and postural control in Parkinson’s patients. Clinically, MBET (SUCRA = 83.1%) can be prioritized to improve gait stabilization, but caution is needed as the gait stabilization outcome exhibits moderate publication bias (Egger test *p* = 0.020), which may overestimate the effect size by 15–20%; No significant publication bias was observed for fall risk and postural control outcomes (Egger test *p* = 0.760, *p* = 0.599), indicating higher evidence robustness. RPT (SUCRA = 97.4%) is the preferred option for reducing fall risk, with its efficacy less affected by bias; PCT (SUCRA = 92.4%) is the most robust option for improving postural control (Egger test *p* = 0.599). Future research recommendations: ① search platforms such as ClinicalTrials.gov to identify unpublished RCTs; ② include non-English studies (e.g., Japanese, Korean literature) to reduce language bias; ③ use the trimming method to correct effect sizes for gait stabilization outcomes.

## Data Availability

The original contributions presented in the study are included in the article/Supplementary material, further inquiries can be directed to the corresponding author.
