# Supplementary material for: A comparative meta-analysis of seven types of exercise-based physical therapy for gait stabilization, fall risk, and postural control in Parkinson’s disease patients
Source: Front Neurol. 2025 Dec 5;16:1706561. doi: 10.3389/fneur.2025.1706561 (PMC12716154; doi:10.3389/fneur.2025.1706561)
Supplement: Supplementary file 1 [file Table_1.DOCX]

**Table 1.** Basic features of the included studies

| Study | Country | Group | Sample size(M/F) | Age (mean ± SD) | Intervention  duration | Intervention  frequency | Outcome measure | Course of disease (year) |
| --- | --- | --- | --- | --- | --- | --- | --- | --- |
| Landers 2016[1] | USA | SSMT | 10**（6/4）** | 72.2 ± 4.4 | 4w | 45min,3 times/week | Gait speed(m/s)、TUG、UPDRS、BBS |  |
|  |  | PCT | 10**（7/3）** | 70.1 ± 9.5 |  | 45min,3 times/week |  |  |
|  |  | RPT | 10**（6/4）** | 74.3 ± 8.8 |  |  |  |  |
| Calabrò 2019[2] | Italy | SSMT | 25(9/11) | 70 ± 8 | 8w | 30min,5 times/week | TUG、Gait velocity、UPDRS、BBS | 10 ± 3 |
|  |  | AE | 25(6/14) | 73 ± 8 |  | 30min,5 times/week |  | 9.3 ± 3 |
| Capato 2020[3] | Brazil | SSMT | 56**（27/29）** | 74 ± 8 | 5w | 45min,twice/week | TUG-MAN、BBS |  |
|  |  | PCT | 50**（18/32）** | 67 ± 13 |  | 45min,twice/week |  |  |
|  |  | RPT | 48**（19/29）** | 73 ± 10 | 24±3 |  |  |  |
| Carpinella 2017[4] | Italy | SSMT | 17**（3/14）** | 73.0 ± 7.1 | 7w | 45min,3 times/week | TUG、Gait velocity、UPDRS | 7.5 ± 3.2 |
|  |  | RPT | 20**（11/9）** | 75.6 ± 8.2 |  | 45min,3 times/week |  | 10.3 ± 5.7 |
| Cherup 2021[5] | USA | MBET | 15(5/10) | 69.8 ± 7.3 | 12w | 45min,twice/week | TUG (s)、BBS、Gait velocity |  |
|  |  | PCT | 18(7/11) | 71.4 ± 12.1 |  | 45min,twice/week |  |  |
| Çoban 2021[6] | Turkey | MBET | 20(11/9) | 58.85 ± 8.09 | 8w | 45min,twice/week | TUG、Gait velocity、UPDRS | 5.32 ± 6.23 |
|  |  | RPT | 20(10/10) | 60.75 ± 7.62 |  | 45min,twice/week |  | 5.32 ± 6.23 |
| Conradsson 2015[7] | Sweden | PCT | 47**（19/28）** | 72.9 ± 6.0 | 10w | 60min,3 times/week | Gait speed(m/s)、TUG、UPDRS | 6.0 ± 5.1 |
|  |  | RPT | 45**（22/23）** | 73.6 ± 5.3 |  | 60min,3 times/week |  | 5.6 ± 5.0 |
| Hackney 2007[8] | USA | MBET | 9(3/6) | 72.6 ± 2.20 | 13w | 60min,twice/week | Gait speed(m/s)、UPDRS、BBS | 6.2 ± 1.5 |
|  |  | RTRT | 10(4/6) | 69.6 ± 2.1 |  | 60min,twice/week |  | 3.3 ± 0.5 |
| Han 2023[9] | China | SSMT | 24(12/12) | 58.17 ± 5.36 | 4w | 20min,5 times/week | TUG、Gait velocity、UPDRS | 1.71 ± 0.75 |
|  |  | RPT | 24(13/11) | 56.25 ± 4.82 |  | 30min,5 times/week |  | 1.83 ± 0.70 |
| Picelli 2012[10] | Italy | AE | 17 | 68.3 ± 7.5 | 4w | 40min,3 times/week | UPDRS 、TUG、Gait velocity |  |
|  |  | RPT | 17 | 68.3 ± 7.5 |  | 40min,3 times/week |  |  |
| Ni 2016[11] | USA | RTRT | 14(5/9) | 71.6 ± 6.6 | 12w | 60min,twice/week | BBS、Gait velocity、UPDRS | 6.6 ± 4.4 |
|  |  | MBET | 13(2/11) | 71.2 ± 6.5 |  | 60min,twice/week |  | 6.9 ± 6.3 |
|  |  | RPT | 12(6/6) | 74.9 ± 8.3 |  |  |  |  |
| Smania 2010[12] | Italy | PCT | 28**（14/14）** | 67.64 ± 7.41 | 7w | 50min,3 times/week | UPDRS 、Gait velocity、TUG | 10.39 ± 4.76 |
|  |  | RPT | 27**（15/12）** | 67.26 ± 7.18 |  | 50min,3 times/week |  | 8.63 ± 5.39 |
| Steib 2017[13] | Germany | PCT | 18(7/11) | 67.6 ± 8.2 | 8w | 40min,twice/week | Gait speed(m/s)、TUG、BBS | 7.9 ± 4.0 |
|  |  | AE | 19(4/15) | 62.5 ± 7.9 |  | 40min,twice/week |  | 7.3 ± 4.4 |
| Van 2018[14] | USA | MBET | 15(10/5) | 65.53 ± 6.09 | 8w | 60min,twice/week | sMini-BESTest、TUG、UPDRS |  |
|  |  | RPT | 12(7/5) | 70.5 ± 4.44 |  |  |  |  |
| Wallén 2018[15] | Sweden | RTRT | 51**（19/32)** | 73.1 ± 5.8 | 10w | 60min,3 times/week | Gait speed(m/s)、TUG、UPDRS | 5.9 ± 5.1 |
|  |  | RPT | 49**（24/25)** | 73.0 ± 5.5 |  |  |  | 5.6 ± 4.8 |
| Xiao 2016[16] | China | TCRT | 45(14/31) | 68.17 ± 2.27 | 6m | 45min,4 times/week | Gaitspeed(m/s)、UPDRS、BBS | 5.45 ± 3.61 |
|  |  | AE | 44(13/31) | 66.52 ± 2.13 |  | 30min,7 times/week |  | 6.15 ± 2.63 |
| Zhao 2024[17] | China | RPT | 24(11/13) | 68.58 ± 10.05 | 4 | 30min,twice/day,5 times/week | BBS、Gait velocity、TUG、UPDRS | 4.96 ± 1.55 |
|  |  | SSMT | 24(10/14) | 67.92 ± 6.82 |  | 30min,twice/day,5 times/week |  | 5.17 ± 1.74 |
| Mak 2021[18] | China | PCT | 33**（22/11）** | 61.9 ± 6.4 | 6w | 90min,once/week | Gait speed(m/s)、TUG、UPDRS | 5.8 ± 6.0 |
|  |  | RTRT | 31**（22/9)** | 62.7 ± 7.2 |  | 3 times/week |  | 5.0 ± 4.0 |
| Bello 2013[19] | Spain | AE | 11**（4/7)** | 59.45 ± 11.32 | 5w | 3 times/week | maximal speed、TUG、UPDRS | 4.82 ± 3.28 |
|  |  | PCT | 11**（5/6)** | 58.00 ± 9.38 |  |  |  | 4.95 ± 2.59 |
| Cardalda 2018[20] | Spain | MBET | 13(7/6) | 62.85 ± 9.75 | 12w | 60min,twice/week | TUG、Gait velocity、UPDRS |  |
|  |  | AE | 13(8/5) | 66.00 ± 13.14 |  | 60min,twice/week |  |  |
| Landers 2016[21] | China | MBET | 71**（34/37）** | 63.7 ± 8.2 | 8w | 90min,once/week | Gait speed(m/s)、UPDRS、BBS |  |
|  |  | RTRT | 67**（39/28）** | 63.5 ± 9.3 |  | 90min,once/week |  |  |
| Li 2022[22] | China | TCRT | 32**（15/17）** | 62.7 ± 5.51 | 12m | 60min,3 times/week | Gait speed(m/s)、TUG、UPDRS | 5.91 ± 4.01 |
|  |  | AE | 31**（9/22)** | 61.9 ± 5.64 |  | 60min,3 times/week |  | 3.82 ± 1.87 |
|  |  | RPT | 32**（13/19）** | 61.9 ± 6.76 |  | 60min,3 times/week |  | 4.32 ± 2.46 |
| Zhang 2015[23] | China | TCRT | 20**（7/13）** | 66.00 ± 11.80 | 12w | 60min,twice/week | Gait velocity, cm/sec、TUG、UPDRS | 6.80 ± 5.43 |
|  |  | AE | 20**（9/11）** | 64.35 ± 10.53 |  | 60min,twice/week |  | 4.85 ± 3.72 |
| Hackney 2008[24] | USA | TCRT | 13(2/11) | 64.9 ± 8.3 | 10w | 60min,twice/week | Velocity、TUG、UPDRS | 8.7 ± 4.7 |
|  |  | RPT | 13(3/10) | 62.6 ± 10.2 |  |  |  | 5.5 ± 3.3 |
| Sage 2009[25] | Canada | SSMT | 18(6/12) | 64.2 ± 10.3 | 12w | 40-60min,3 times/week | Gait speed(m/s)、TUG、UPDRS | 4.7 ± 4.9 |
|  |  | AE | 13(7/6) | 65.1 ± 9.3 |  | 30min,3 times/week |  | 3.2 ± 2.9 |
|  |  | RPT | 15**（7/8）** | 68.6 ± 8.7 |  |  |  | 2.5 ± 2.2 |
| Schlick 2016[26] | Germany | SSMT | 10**（8/2）** | 71.2 ± 10.9 | 5w | 20-45min,3 times/week | Gait speed, km/h、UPDRS | 10.4 ± 5.2 |
|  |  | AE | 10**（6/4）** | 68.9 ± 6.8 |  | 20-45min,3 times/week |  | 9.1 ± 3.1 |
| San 2020[27] | Spain | AE | 23**（12/11)** | 66.38 ± 7.06 | 10w | 60min,twice/week | Gait speed(m/s)、TUG、UPDRS |  |
|  |  | PCT | 17**（5/12）** | 64.75 ± 8.77 |  | 60min,twice/week |  |  |
| Faria 2023[28] | Brazil | MBET | 10(5/5) | 62.2 ± 10.6 | 20w | 60min,3 times/week | Gait speed(m/s)、TUG、UPDRS | 6.3 ± 4 |
|  |  | AE | 12(6/6) | 64.8 ± 8.9 |  | 60min,3 times/week |  | 8.1 ± 3.2 |
| Amano 2013[29] | USA | TCRT | 12(5/7) | 64 ± 13 | 16w | 60min,3 times/week | Gaitvelocity(m/s)、UPDRS | 7 ± 7 |
|  |  | MBET | 9**（2/7）** | 68 ± 7 |  | 60min,3 times/week |  | 12 ± 7 |
|  |  | RPT | 15**（8/7）** | 66 ± 11 |  |  |  |  |
| Frazzitta 2009[30] | Italy | SSMT | 20**（12/8）** | 71 ± 8 | 4w | 20min,twice/week | Gait speed(m/s)、UPDRS、BBS | 13.2 ± 4.1 |
|  |  | AE | 20**（11/9）** | 71 ± 7 |  | 20min,twice/week |  | 12.9 ± 4.6 |
| Shen 2012[31] | China | SSMT | 14(5/9) | 63.0 ± 8.5 | 4w | 45min,3 times/week | Gait velocity (cm/s)、UPDRS、BBS | 7.1 ± 3.2 |
|  |  | RTRT | 14(7/7) | 66.5 ± 8.6 |  | 60min,3 times/week |  | 5.8 ± 2.2 |
| Zhou 2024[32] | China | MBET | 14**（6/8）** | 62.29 ± 5.37 | 8w | 90min,twice/week | Gait speed(m/s)、UPDRS、BBS | 6.41 ± 5.14 |
|  |  | AE | 14**（5/9）** | 63.14 ± 9.39 |  | 90min,twice/week |  | 4.09 ± 3.92 |
| Li 2012[33] | China | TCRT | 65(20/45) | 68 ± 9 | 24w | 60min,twice/week | Gait velocity (cm/sec)、TUG、UPDRS | 8 ± 9 |
|  |  | RTRT | 65(27/38) | 69 ± 8 |  | 60min,twice/week |  | 8 ± 9 |
|  |  | RPT | 65(26/39) | 69 ± 9 |  | 60min,twice/week |  |  |
| Wong-Yu 2015[34] | China | PCT | 32**（13/19）** | 60.2 ± 9.0 | 8w | 120min,once/week | Gaitspeed(m/s)、UPDRS | 7.3 ± 4.6 |
|  |  | RTRT | 36**（16/20）** | 61.9 ± 8.5 |  | 120min,once/week |  | 5.4 ± 3.6 |
| Nadeau 2014[35] | Canada | SSMT | 12**（4/8）** | 64.0 ± 6.6 | 24w | 60min,3 times/week | UPDRS、TUG |  |
|  |  | AE | 11**（2/9）** | 64.3 ± 5.6 |  | 60min,twice/week |  |  |
| Duncan 2012[36] | USA | MBET | 26(11/15) | 69.3 ± 1.9 | 12m | 60min,twice/week | Gait speed(m/s)、UPDRS | 5.8 ± 1.1 |
|  |  | RPT | 26(11/15) | 69.0 ± 1.5 |  |  |  | 7.0 ± 1.0 |
| Hashimoto 2015[37] | Japan | MBET | 15(12/3) | 67.9 ± 7.0 | 12w | 60min,once/week | UPDRS、Gait velocity、TUG | 6.3 ± 4.6 |
|  |  | AE | 17**（15/2）** | 62.7 ± 14.9 |  | 60min,once/week |  | 7.8 ± 6.2 |
|  |  | RPT | 14(7/7) | 69.7 ± 4.0 |  |  |  | 6.9 ± 4.0 |
| Solla 2019[38] | Italy | MBET | 10(4/6) | 67.8 ± 5.9 | 12w | 90min,twice/week | Gait speed(m/s) | 4.4 ± 4.5 |
|  |  | CRPT | 10**（3/7）** | 67.1 ± 6.3 |  |  |  | 5 ± 2.9 |
| Frisaldi 2021[39] | Italy | MBET | 19(9/10) | 60.68 ± 6.34 | 5w | 120min,3 times/week | Mini-BESTest、TUG、UPDRS | 5.99 ± 2.18 |
|  |  | RPT | 19(6/13) | 61.21 ± 7.18 |  | 120min,3 times/week |  | 6.43 ± 2.50 |
| Romenets 2015[40] | Canada | MBET | 18(6/12) | 63.2 ± 9.9 | 12w | 60min,twice/week | mini-BESTest、UPDRS | 5.5 ± 4.4 |
|  |  | RPT | 15(8/7) | 64.3 ± 8.1 |  |  |  | 7.7 ± 4.6 |
| Fatollahkhani 2019[41] | Iran | AE | 11**（3/8）** | 60.63 ± 9.36 | 10w | 30min,twice/week | TUG、Gait velocity、UPDRS、BBS | 8.89 ± 5.14 |
|  |  | RPT | 9**（2/7）** | 61.55 ± 8.57 |  |  |  | 8.50 ± 6.34 |
| Fisher 2008[42] | USA | AE | 10**（4/6）** | 64.0 ± 14.5 | 8w | 45min,3 times/week | Gait speed(m/s)、TUG、UPDRS | 14.7 ± 9.9 |
|  |  | RPT | 10**（5/5）** | 61.5 ± 9.8 |  | 45min,3 times/week |  | 8.8 ± 7.9 |
|  |  | AE | 10**（2/8）** | 63.1 ± 11.5 |  | 60min,once/week |  | 17.7 ± 13.3 |
| Gaßner 2022[43] | Germany | AE | 49**（12/37)** | 60.5 ± 9.1 | 12w | 25min,5 times/week | Velocity in m/s、TUG |  |
|  |  | RPT | 51**（14/37)** | 61.7 ± 8.1 |  | 25min,5 times/week |  |  |
| Jaywant 2016[44] | USA | AE | 13(7/6) | 63.7 ± 6.2 | 8day | everyday | Walking Speed  、TUG、UPDRS |  |
|  |  | RPT | 10**（6/4）** | 65.8 ± 8.7 |  |  |  |  |
| Skrzatek 2024[45] | France | RTRT | 11 | 71.1 ± 6.2 | 6w | 3 times/week | Gait speed(m/s)、BBS | 13.1 ± 5.3 |
|  |  | CRT | 12 | 65.1 ± 6.2 |  | 3 times/week |  | 13.5 ± 7.6 |
| Chang 2024[46] | China | TCRT | 16(9/7) | 66.31 ± 6.54 | 12w | 60min,3 times/week | UPDRS、BBS | 6.75 ± 5.49 |
|  |  | AE | 14(9/5) | 64.43 ± 7.37 |  | 30min,3 times/week |  | 6.57 ± 7.92 |
|  |  | RPT | 13(7/6) | 63.15 ± 7.95 |  |  |  | 6.77 ± 6.84 |
| Gryfe 2022[47] | Canada | RPT | 14(7/7) | 70.7 ± 7.3 | 8w | 60min,twice/week | UPDRS、Gait velocity |  |
|  |  | AE | 13(3/10) | 69.3 ± 8.0 |  |  |  |  |
| Marusiak 2019[48] | Brazil | AE | 10**（3/7）** | 72 ± 10 | 8w | 60min,3 times/week | UPDRS、BBS | 9 ± 5 |
|  |  | RPT | 10**（4/6）** | 74 ± 9 |  |  |  | 8 ± 4 |
| Picelli 2013[49] | Italy | AE | 20(14/6) | 68.80 ± 7.72 | 4w | 45min,3 times/week | UPDRS | 6.99 ± 6.17 |
|  |  | RPT | 20**（12/8）** | 67.55 ± 7.08 |  | 30min,3 times/week |  | 6.79 ± 6.30 |
| Picelli 2016[50] | Italy | AE | 9(4/5) | 71.2 ± 9.2 | 4w | 45min,3 times/week | UPDRS 、TUG | 11.2 ± 5.6 |
|  |  | RPT | 8(4/4) | 71.6 ± 7.2 |  |  |  | 10.8 ± 4.1 |
| Schenkman 2018[51] | USA | AE | 45**（27/18)** | 63 ± 10 | 4w | 4 times/week | UPDRS 、Gait velocity | 1.5 ± 3.1 |
|  |  | RPT | 40**（24/16)** | 64 ± 10 |  |  |  | 1.4 ± 2.1 |
| Furnari 2017[52] | Italy | SSMT | 19(8/11) | 71.5 ± 11.7 | 4w | 60min,6 times/week | UPDRS、TUG |  |
|  |  | PCT | 19(9/10) | 77.7 ± 8.3 |  | 60min,6 times/week |  |  |
| Zhang 2023[53] | China | SSMT | 32(13/19) | 63.87 ± 5.60 | 8w | 40min, 5 times/week | UPDRS 、Gait velocity | 4.23 ± 0.79 |
|  |  | RPT | 32(15/17) | 64.03 ± 5.28 |  | 50min, 5 times/week |  | 4.50 ± 0.82 |
| Shen 2014[54] | China | SSMT | 22（9/13） | 63.3 ± 8.0 | 12w | 60min,3-5 times/week | Gait velocity (cm/s)、TUG、UPDRS | 8.1 ± 4.3 |
|  |  | RTRT | 23（11/12) | 65.3 ± 8.5 |  | 60min,3-5 times/week |  | 6.6 ± 4.0 |

1. Landers, M. R., Hatlevig, R. M., Davis, A. D., Richards, A. R., & Rosenlof, L. E. (2016). Does attentional focus during balance training in people with Parkinson's disease affect outcome? A randomised controlled clinical trial. Clinical rehabilitation, 30(1), 53–63. <https://doi.org/10.1177/0269215515570377>
2. Calabrò, R. S., Naro, A., Filoni, S., Pullia, M., Billeri, L., Tomasello, P., Portaro, S., Di Lorenzo, G., Tomaino, C., & Bramanti, P. (2019). Walking to your right music: a randomized controlled trial on the novel use of treadmill plus music in Parkinson's disease. Journal of neuroengineering and rehabilitation, 16(1), 68. <https://doi.org/10.1186/s12984-019-0533>
3. Capato, T. T. C., de Vries, N. M., IntHout, J., Barbosa, E. R., Nonnekes, J., & Bloem, B. R. (2020). Multimodal Balance Training Supported by Rhythmical Auditory Stimuli in Parkinson's Disease: A Randomized Clinical Trial. Journal of Parkinson's disease, 10(1), 333–346. <https://doi.org/10.3233/JPD-191752>
4. Carpinella, I., Cattaneo, D., Bonora, G., Bowman, T., Martina, L., Montesano, A., & Ferrarin, M. (2017). Wearable Sensor-Based Biofeedback Training for Balance and Gait in Parkinson Disease: A Pilot Randomized Controlled Trial. Archives of physical medicine and rehabilitation, 98(4), 622–630.e3. <https://doi.org/10.1016/j.apmr.2016.11.003>
5. Cherup, N. P., Strand, K. L., Lucchi, L., Wooten, S. V., Luca, C., & Signorile, J. F. (2021). Yoga Meditation Enhances Proprioception and Balance in Individuals Diagnosed With Parkinson's Disease. Perceptual and motor skills, 128(1), 304–323. <https://doi.org/10.1177/0031512520945085>
6. Çoban, F., Belgen Kaygısız, B., & Selcuk, F. (2021). Effect of clinical Pilates training on balance and postural control in patients with Parkinson's disease: a randomized controlled trial. Journal of comparative effectiveness research, 10(18), 1373–1383. <https://doi.org/10.2217/cer-2021-0091>
7. Conradsson 2015
8. Hackney, M. E., Kantorovich, S., Levin, R., & Earhart, G. M. (2007). Effects of tango on functional mobility in Parkinson's disease: a preliminary study. Journal of neurologic physical therapy : JNPT, 31(4), 173–179. <https://doi.org/10.1097/NPT.0b013e31815ce78b>
9. Han, T., Liu, Q., Hu, Y., Wang, Y., & Xue, K. (2023). Effect of Pro-kin visual feedback balance training on balance function of individuals with early Parkinson's disease: a randomized controlled pilot trial. African health sciences, 23(2), 582–588. <https://doi.org/10.4314/ahs.v23i2.67>
10. Picelli, A., Melotti, C., Origano, F., Waldner, A., Gimigliano, R., & Smania, N. (2012). Does robotic gait training improve balance in Parkinson's disease? A randomized controlled trial. Parkinsonism & related disorders, 18(8), 990–993. <https://doi.org/10.1016/j.parkreldis.2012.05.010>
11. Ni, M., Mooney, K., & Signorile, J. F. (2016). Controlled pilot study of the effects of power yoga in Parkinson's disease. Complementary therapies in medicine, 25, 126–131. <https://doi.org/10.1016/j.ctim.2016.01.007>
12. Smania, N., Corato, E., Tinazzi, M., Stanzani, C., Fiaschi, A., Girardi, P., & Gandolfi, M. (2010). Effect of balance training on postural instability in patients with idiopathic Parkinson's disease. Neurorehabilitation and neural repair, 24(9), 826–834. <https://doi.org/10.1177/1545968310376057>
13. Steib, S., Klamroth, S., Gaßner, H., Pasluosta, C., Eskofier, B., Winkler, J., Klucken, J., & Pfeifer, K. (2017). Perturbation During Treadmill Training Improves Dynamic Balance and Gait in Parkinson's Disease: A Single-Blind Randomized Controlled Pilot Trial. Neurorehabilitation and neural repair, 31(8), 758–768. <https://doi.org/10.1177/1545968317721976>
14. Van Puymbroeck, M., Walter, A. A., Hawkins, B. L., Sharp, J. L., Woschkolup, K., Urrea-Mendoza, E., Revilla, F., Adams, E. V., & Schmid, A. A. (2018). Functional Improvements in Parkinson's Disease Following a Randomized Trial of Yoga. Evidence-based complementary and alternative medicine : eCAM, 2018, 8516351. <https://doi.org/10.1155/2018/8516351>
15. Wallén, M. B., Hagströmer, M., Conradsson, D., Sorjonen, K., & Franzén, E. (2018). Long-term effects of highly challenging balance training in Parkinson's disease-a randomized controlled trial. Clinical rehabilitation, 32(11), 1520–1529. <https://doi.org/10.1177/0269215518784338>
16. Xiao, C. M., & Zhuang, Y. C. (2016). Effect of health Baduanjin Qigong for mild to moderate Parkinson's disease. Geriatrics & gerontology international, 16(8), 911–919. <https://doi.org/10.1111/ggi.12571>
17. Zhao, W., Li, Y., You, H., Feng, X., & Lei, Y. (2024). Effects of repetitive transcranial magnetic stimulation combined with visual feedback balance training on balance and gait in patients with Parkinson's disease. Chinese Journal of Rehabilitation Medicine, 39(9), 1327-1331.
18. Mak, M. K. Y., & Wong-Yu, I. S. K. (2021). Six-Month Community-Based Brisk Walking and Balance Exercise Alleviates Motor Symptoms and Promotes Functions in People with Parkinson's Disease: A Randomized Controlled Trial. Journal of Parkinson's disease, 11(3), 1431–1441. <https://doi.org/10.3233/JPD-202503>
19. Bello, O., Sanchez, J. A., Lopez-Alonso, V., Márquez, G., Morenilla, L., Castro, X., Giraldez, M., Santos-García, D., & Fernandez-del-Olmo, M. (2013). The effects of treadmill or overground walking training program on gait in Parkinson's disease. Gait & posture, 38(4), 590–595. <https://doi.org/10.1016/j.gaitpost.2013.02.005>
20. Mollinedo-Cardalda, I., Cancela-Carral, J. M., & Vila-Suárez, M. H. (2018). Effect of a Mat Pilates Program with TheraBand on Dynamic Balance in Patients with Parkinson's Disease: Feasibility Study and Randomized Controlled Trial. Rejuvenation research, 21(5), 423–430. <https://doi.org/10.1089/rej.2017.2007>
21. Landers, M. R., Hatlevig, R. M., Davis, A. D., Richards, A. R., & Rosenlof, L. E. (2016). Does attentional focus during balance training in people with Parkinson's disease affect outcome? A randomised controlled clinical trial. Clinical rehabilitation, 30(1), 53–63. <https://doi.org/10.1177/0269215515570377>
22. Li, G., Huang, P., Cui, S. S., Tan, Y. Y., He, Y. C., Shen, X., Jiang, Q. Y., Huang, P., He, G. Y., Li, B. Y., Li, Y. X., Xu, J., Wang, Z., & Chen, S. D. (2022). Mechanisms of motor symptom improvement by long-term Tai Chi training in Parkinson's disease patients. Translational neurodegeneration, 11(1), 6. <https://doi.org/10.1186/s40035-022-00280-7>
23. Zhang, T. Y., Hu, Y., Nie, Z. Y., Jin, R. X., Chen, F., Guan, Q., Hu, B., Gu, C. Y., Zhu, L., & Jin, L. J. (2015). Effects of Tai Chi and Multimodal Exercise Training on Movement and Balance Function in Mild to Moderate Idiopathic Parkinson Disease. American journal of physical medicine & rehabilitation, 94(10 Suppl 1), 921–929. <https://doi.org/10.1097/PHM.0000000000000351>
24. Hackney, M. E., & Earhart, G. M. (2008). Tai Chi improves balance and mobility in people with Parkinson disease. Gait & posture, 28(3), 456–460. <https://doi.org/10.1016/j.gaitpost.2008.02.005>
25. Sage, M. D., & Almeida, Q. J. (2009). Symptom and gait changes after sensory attention focused exercise vs aerobic training in Parkinson's disease. Movement disorders : official journal of the Movement Disorder Society, 24(8), 1132–1138. <https://doi.org/10.1002/mds.22469>
26. Schlick, C., Ernst, A., Bötzel, K., Plate, A., Pelykh, O., & Ilmberger, J. (2016). Visual cues combined with treadmill training to improve gait performance in Parkinson's disease: a pilot randomized controlled trial. Clinical rehabilitation, 30(5), 463–471. <https://doi.org/10.1177/0269215515588836>
27. San Martín Valenzuela, C., Moscardó, L. D., López-Pascual, J., Serra-Añó, P., & Tomás, J. M. (2020). Effects of Dual-Task Group Training on Gait, Cognitive Executive Function, and Quality of Life in People With Parkinson Disease: Results of Randomized Controlled DUALGAIT Trial. Archives of physical medicine and rehabilitation, 101(11), 1849–1856.e1. <https://doi.org/10.1016/j.apmr.2020.07.008>
28. de Faria, J., Sousa, L. R., Dorásio, A. C. P., Pereira, M. P., Moraes, R., Crozara, L. F., & Hallal, C. Z. (2023). Multicomponent and mat Pilates training increased gait speed in individuals with Parkinson's disease when walking and carrying a load: A single-blinded randomized controlled trial. Physiotherapy research international : the journal for researchers and clinicians in physical therapy, 28(4), e2031. <https://doi.org/10.1002/pri.2031>
29. Amano, S., Nocera, J. R., Vallabhajosula, S., Juncos, J. L., Gregor, R. J., Waddell, D. E., Wolf, S. L., & Hass, C. J. (2013). The effect of Tai Chi exercise on gait initiation and gait performance in persons with Parkinson's disease. Parkinsonism & related disorders, 19(11), 955–960. <https://doi.org/10.1016/j.parkreldis.2013.06.007>
30. Frazzitta, G., Maestri, R., Uccellini, D., Bertotti, G., & Abelli, P. (2009). Rehabilitation treatment of gait in patients with Parkinson's disease with freezing: a comparison between two physical therapy protocols using visual and auditory cues with or without treadmill training. Movement disorders : official journal of the Movement Disorder Society, 24(8), 1139–1143. <https://doi.org/10.1002/mds.22491>
31. Shen, X., & Mak, M. K. (2012). Repetitive step training with preparatory signals improves stability limits in patients with Parkinson's disease. Journal of rehabilitation medicine, 44(11), 944–949. <https://doi.org/10.2340/16501977-1056>
32. Zhou, J. H., Wang, R. Y., Liu, Y. T., Cheng, S. J., Liu, H. H., & Yang, Y. R. (2024). Improving Executive Function and Dual-Task Cost in Parkinson Disease: A Randomized Controlled Trial. Journal of neurologic physical therapy : JNPT, 48(4), 188–197. <https://doi.org/10.1097/NPT.0000000000000489>
33. Li, F., Harmer, P., Fitzgerald, K., Eckstrom, E., Stock, R., Galver, J., Maddalozzo, G., & Batya, S. S. (2012). Tai chi and postural stability in patients with Parkinson's disease. The New England journal of medicine, 366(6), 511–519. <https://doi.org/10.1056/NEJMoa1107911>
34. Wong-Yu, I. S., & Mak, M. K. (2015). Task- and Context-Specific Balance Training Program Enhances Dynamic Balance and Functional Performance in Parkinsonian Nonfallers: A Randomized Controlled Trial With Six-Month Follow-Up. Archives of physical medicine and rehabilitation, 96(12), 2103–2111. <https://doi.org/10.1016/j.apmr.2015.08.409>
35. Nadeau, A., Pourcher, E., & Corbeil, P. (2014). Effects of 24 wk of treadmill training on gait performance in Parkinson's disease. Medicine and science in sports and exercise, 46(4), 645–655. <https://doi.org/10.1249/MSS.0000000000000144>
36. Duncan, R. P., & Earhart, G. M. (2012). Randomized controlled trial of community-based dancing to modify disease progression in Parkinson disease. Neurorehabilitation and neural repair, 26(2), 132–143. <https://doi.org/10.1177/1545968311421614>
37. Hashimoto, H., Takabatake, S., Miyaguchi, H., Nakanishi, H., & Naitou, Y. (2015). Effects of dance on motor functions, cognitive functions, and mental symptoms of Parkinson's disease: a quasi-randomized pilot trial. Complementary therapies in medicine, 23(2), 210–219. <https://doi.org/10.1016/j.ctim.2015.01.010>
38. Solla, P., Cugusi, L., Bertoli, M., Cereatti, A., Della Croce, U., Pani, D., Fadda, L., Cannas, A., Marrosu, F., Defazio, G., & Mercuro, G. (2019). Sardinian Folk Dance for Individuals with Parkinson's Disease: A Randomized Controlled Pilot Trial. Journal of alternative and complementary medicine (New York, N.Y.), 25(3), 305–316. <https://doi.org/10.1089/acm.2018.0413>
39. Frisaldi, E., Bottino, P., Fabbri, M., Trucco, M., De Ceglia, A., Esposito, N., Barbiani, D., Camerone, E. M., Costa, F., Destefanis, C., Milano, E., Massazza, G., Zibetti, M., Lopiano, L., & Benedetti, F. (2021). Effectiveness of a dance-physiotherapy combined intervention in Parkinson's disease: a randomized controlled pilot trial. Neurological sciences : official journal of the Italian Neurological Society and of the Italian Society of Clinical Neurophysiology, 42(12), 5045–5053. <https://doi.org/10.1007/s10072-021-05171-9>
40. Rios Romenets, S., Anang, J., Fereshtehnejad, S. M., Pelletier, A., & Postuma, R. (2015). Tango for treatment of motor and non-motor manifestations in Parkinson's disease: a randomized control study. Complementary therapies in medicine, 23(2), 175–184. <https://doi.org/10.1016/j.ctim.2015.01.015>
41. Arfa-Fatollahkhani, P., Safar Cherati, A., Habibi, S. A. H., Shahidi, G. A., Sohrabi, A., & Zamani, B. (2019). Effects of treadmill training on the balance, functional capacity and quality of life in Parkinson's disease: A randomized clinical trial. Journal of complementary & integrative medicine, 17(1), /j/jcim.2019.17.issue-1/jcim-2018-0245/jcim-2018-0245.xml. <https://doi.org/10.1515/jcim-2018-0245>
42. Fisher, B. E., Wu, A. D., Salem, G. J., Song, J., Lin, C. H., Yip, J., Cen, S., Gordon, J., Jakowec, M., & Petzinger, G. (2008). The effect of exercise training in improving motor performance and corticomotor excitability in people with early Parkinson's disease. Archives of physical medicine and rehabilitation, 89(7), 1221–1229. <https://doi.org/10.1016/j.apmr.2008.01.013>
43. Gaßner, H., Trutt, E., Seifferth, S., Friedrich, J., Zucker, D., Salhani, Z., Adler, W., Winkler, J., & Jost, W. H. (2022). Treadmill training and physiotherapy similarly improve dual task gait performance: a randomized-controlled trial in Parkinson's disease. Journal of neural transmission (Vienna, Austria : 1996), 129(9), 1189–1200. <https://doi.org/10.1007/s00702-022-02514-4>
44. Jaywant, A., Ellis, T. D., Roy, S., Lin, C. C., Neargarder, S., & Cronin-Golomb, A. (2016). Randomized Controlled Trial of a Home-Based Action Observation Intervention to Improve Walking in Parkinson Disease. Archives of physical medicine and rehabilitation, 97(5), 665–673. <https://doi.org/10.1016/j.apmr.2015.12.029>
45. Skrzatek, A., Nuic, D., Cherif, S., Beranger, B., Gallea, C., Bardinet, E., & Welter, M. L. (2024). Brain modulation after exergaming training in advanced forms of Parkinson's disease: a randomized controlled study. Journal of neuroengineering and rehabilitation, 21(1), 133. <https://doi.org/10.1186/s12984-024-01430-w>
46. Chang, C. L., Lin, T. K., Pan, C. Y., Wang, T. C., Tseng, Y. T., Chien, C. Y., & Tsai, C. L. (2024). Distinct effects of long-term Tai Chi Chuan and aerobic exercise interventions on motor and neurocognitive performance in early-stage Parkinson's disease: a randomized controlled trial. European journal of physical and rehabilitation medicine, 60(4), 621–633. <https://doi.org/10.23736/S1973-9087.24.08166-8>
47. Gryfe, P., Sexton, A., & McGibbon, C. A. (2022). Using gait robotics to improve symptoms of Parkinson's disease: an open-label, pilot randomized controlled trial. European journal of physical and rehabilitation medicine, 58(5), 723–737. <https://doi.org/10.23736/S1973-9087.22.07549-9>
48. Marusiak, J., Fisher, B. E., Jaskólska, A., Słotwiński, K., Budrewicz, S., Koszewicz, M., Kisiel-Sajewicz, K., Kamiński, B., & Jaskólski, A. (2019). Eight Weeks of Aerobic Interval Training Improves Psychomotor Function in Patients with Parkinson's Disease-Randomized Controlled Trial. International journal of environmental research and public health, 16(5), 880. <https://doi.org/10.3390/ijerph16050880>
49. Picelli, A., Melotti, C., Origano, F., Neri, R., Waldner, A., & Smania, N. (2013). Robot-assisted gait training versus equal intensity treadmill training in patients with mild to moderate Parkinson's disease: a randomized controlled trial. Parkinsonism & related disorders, 19(6), 605–610. <https://doi.org/10.1016/j.parkreldis.2013.02.010>
50. Picelli, A., Varalta, V., Melotti, C., Zatezalo, V., Fonte, C., Amato, S., Saltuari, L., Santamato, A., Fiore, P., & Smania, N. (2016). Effects of treadmill training on cognitive and motor features of patients with mild to moderate Parkinson's disease: a pilot, single-blind, randomized controlled trial. Functional neurology, 31(1), 25–31. <https://doi.org/10.11138/fneur/2016.31.1.025>
51. Schenkman, M., Moore, C. G., Kohrt, W. M., Hall, D. A., Delitto, A., Comella, C. L., Josbeno, D. A., Christiansen, C. L., Berman, B. D., Kluger, B. M., Melanson, E. L., Jain, S., Robichaud, J. A., Poon, C., & Corcos, D. M. (2018). Effect of High-Intensity Treadmill Exercise on Motor Symptoms in Patients With De Novo Parkinson Disease: A Phase 2 Randomized Clinical Trial. JAMA neurology, 75(2), 219–226. <https://doi.org/10.1001/jamaneurol.2017.3517>
52. Furnari, A., Calabrò, R. S., De Cola, M. C., Bartolo, M., Castelli, A., Mapelli, A., Buttacchio, G., Farini, E., Bramanti, P., & Casale, R. (2017). Robotic-assisted gait training in Parkinson's disease: a three-month follow-up randomized clinical trial. The International journal of neuroscience, 127(11), 996–1004. <https://doi.org/10.1080/00207454.2017.1288623>
53. Zhang, L., Gao, L., Xue, C., Zhao, Y., Zhao, R., & Liu, A. (2023). Clinical efficacy of virtual reality technology combined with repetitive transcranial magnetic stimulation in the treatment of mild cognitive impairment in Parkinson's disease. Chinese Journal of Rehabilitation, 38(3), 148-152.
54. Shen, X., & Mak, M. K. (2014). Balance and Gait Training With Augmented Feedback Improves Balance Confidence in People With Parkinson's Disease: A Randomized Controlled Trial. Neurorehabilitation and neural repair, 28(6), 524–535. https://doi.org/10.1177/1545968313517752
