# Supplementary material for: A comparative meta-analysis of seven types of exercise-based physical therapy for gait stabilization, fall risk, and postural control in Parkinson’s disease patients
Source: Front Neurol. 2025 Dec 5;16:1706561. doi: 10.3389/fneur.2025.1706561 (PMC12716154; doi:10.3389/fneur.2025.1706561)
Supplement: Supplementary file 2 [file Table_2.DOCX]

| **Intervention Category (Abbreviation)** | **Core Definition & Classification Criteria** | **Representative Exercise Programs / Examples** | **Primary Training Focus & Emphasis** | **Key Differentiators from Similar Categories** |
| --- | --- | --- | --- | --- |
| **Routine Physical Training (RPT)** | **Standardized, comprehensive foundational exercise therapy**, typically serving as conventional care or an active control in studies. Content is based on evidence-based guidelines covering fundamental motor skills. | • Basic balance training (e.g., weight-shifting) • Gait training (e.g., over-ground walking, obstacle crossing) • Range of motion exercises • Standard stretching | **Comprehensiveness, Foundational.** Aims to maintain and improve basic motor function; serves as the "basic package" for all exercise interventions. | **vs. PCT:** RPT is a general program, while PCT is a **targeted, specialized** balance intervention. **vs. AE:** RPT does not emphasize achieving a specific cardiovascular intensity. |
| **Aerobic Training (AE)** | **Exercise focused on improving cardiorespiratory function and whole-body endurance.** Involves moderate-intensity, rhythmic activities, often targeting 60-70% of maximum heart rate. | • Brisk walking or treadmill training • Stationary cycling • Swimming • Nordic walking | **Cardiovascular Health, Endurance.** Improves motor symptoms indirectly by enhancing overall fitness and reducing fatigue. | The **core criterion is heart rate/endurance target**. Its mechanism for improving motor symptoms is indirect and supportive, unlike more targeted therapies. |
| **Postural Control Training (PCT)** | **Targeted training specifically for static and dynamic balance.** Often challenges the postural control system using biofeedback, perturbations, or unstable surfaces. | • Standing on foam pads or balance boards • Balance reaction training to external pushes • Standing with eyes closed • Training with biofeedback balance systems | **Targeted, Specialized Balance Function.** Directly addresses core deficits in postural stability and sway in PD. | The **core is "challenging the balance system" directly.** It is more specific and demanding than basic balance exercises within RPT. |
| **Mind-Body Exercise Training (MBET)** | **Training systems that emphasize the integration of physical movement with mental focus** (e.g., on breath, meditation, mindfulness). The core principle is regulating motor control and body awareness through mental engagement. | • **Yoga** (e.g., Hatha, Iyengar) • **Pilates** (especially mat-based) • Qigong (certain styles) | **Mind-Body Integration, Proprioception, Cognitive-Motor Coupling.** Aims to optimize movement quality by enhancing mind-body connection, core stability, and psychological state. | **vs. TCRT:** MBET originates from global mind-body philosophies and **does not necessarily incorporate martial arts or Traditional Chinese Medicine theory**; it emphasizes the combination of postures, breath, and mindfulness. **vs. PCT:** MBET improves balance via a mind-body pathway, while PCT does so through direct balance challenges. |
| **Traditional Chinese Rehabilitation Training (TCRT)** | **Mind-body practices rooted in Chinese traditional philosophy** (e.g., Taoism, Traditional Chinese Medicine). Characterized by slow, flowing, circular movements and an emphasis on mental intention guiding "Qi" (vital energy). | • **Tai Chi** (e.g., Yang, Chen styles) • **Baduanjin** (Eight-Section Brocade) • **Wuqinxi** (Five-Animal Frolics) | **Harmony between Motion and Stillness, Unity of Mind and Body.** Improves coordination, gait, and balance through unique movement patterns (e.g., slow, continuous weight transfer). | **vs. MBET:** TCRT has a **distinct cultural origin and theoretical framework (e.g., Yin-Yang, meridians)**. Although both are mind-body practices, their movement paradigms and cultural contexts differ significantly from Yoga/Pilates. This is the primary rationale for its separate classification. |
| **Sensory Stimulation Motor Training (SSMT)** | **Training that uses external cues (auditory, visual, tactile) to guide, initiate, and optimize movement.** It bypasses impaired basal ganglia circuitry by leveraging alternative neural pathways. | • **Gait training guided by a metronome or music** • Stepping over visual markers on the floor • Using vibrotactile cues | **Movement Initiation, Rhythmicity, External Dependency.** Directly addresses "freezing of gait" and rhythmic deficits by compensating for internal timing generation with external sensory input. | The **core is the "use of external sensory stimulation"** to drive movement. It is the only category primarily reliant on external devices/cues, representing a unique mechanism of action. |
| **Resistance Training Rehabilitation Therapy (RTRT)** | **Training involving muscle contractions against external resistance to increase muscular strength, power, and endurance.** Typically follows the principle of progressive overload. | • Resistance band exercises • Machine-based strength training (e.g., leg press, lat pulldown) • Free-weight training (e.g., dumbbell squats) | **Muscle Strength, Power, Hypertrophy.** Provides the necessary musculoskeletal support for postural stability and all motor functions; a foundational element for mobility. | The goal is purely to enhance **"muscle strength"**. While it indirectly improves balance and gait, its direct target is the muscle itself, not the movement pattern or balance perception. |
