# Supplementary material for: A comparative meta-analysis of seven types of exercise-based physical therapy for gait stabilization, fall risk, and postural control in Parkinson’s disease patients
Source: Front Neurol. 2025 Dec 5;16:1706561. doi: 10.3389/fneur.2025.1706561 (PMC12716154; doi:10.3389/fneur.2025.1706561)
Supplement: Supplementary file 3 [file Table_3.DOCX]

**Synthesis of Clinical Constructs into Summary Metrics**

| Construct | Included Outcome Measures | Description & Rationale for Inclusion | Method for Standardization & Integration into a Single Summary Metric |
| --- | --- | --- | --- |
| **Gait Stability** | • **Gait Speed** (e.g., from 10MWT) • **Step Length Variability** (Coefficient of Variation) • **Stride Time Variability** (Coefficient of Variation) • **Double Support Time** | This construct quantifies the smoothness, rhythm, and predictability of walking. **Gait Speed** is a global indicator. **Variability measures** (Step Length, Stride Time) are direct biomarkers of dynamic stability, with higher variability indicating instability. **Double Support Time** increases to improve stability. | **Method: Z-Score Composite Index** 1. **Standardize Scores:** Calculate Z-scores for each measure for every participant: Z = (Individual Score - Sample Mean) / Sample Standard Deviation. 2. **Set Polarity:** Ensure all Z-scores are oriented so that **higher values indicate better stability**. This typically requires inverting Z-scores for variability measures and double support time (e.g., Z_stability = -Z_variability). 3. **Averaging:** Calculate the composite score for each participant as the mean of the standardized, polarity-corrected Z-scores: **Gait Stability Index = Mean(Z_Speed + Z_StepLengthStability + Z_StrideTimeStability + Z_DoubleSupportStability)**. |
| **Fall Risk** | • **Timed Up and Go (TUG)** • **Berg Balance Scale (BBS)** • **Five Times Sit-to-Stand (5TSTS)** • **History of Previous Falls** (self-reported) | This is a multifactorial construct assessing functional mobility, balance, strength, and fall history. **TUG** and **5TSTS** assess dynamic and transitional mobility. **BBS** is a gold-standard for functional balance. **Previous Falls** is the strongest predictor of future falls. | **Method: Risk-Weighted Z-Score Composite** 1. **Standardize Scores:** Calculate Z-scores for TUG, BBS, and 5TSTS. 2. **Set Polarity:** Orient all Z-scores so that **higher values indicate higher fall risk**. Invert BBS (e.g., Z_Risk_BBS = -Z_BBS). TUG and 5TSTS Z-scores already correlate positively with risk. 3. **Incorporate Fall History:** Code previous falls (e.g., 0 for none, 1 for one or more) and convert this to a Z-score. 4. **Combine:** Calculate a weighted average. Fall history can be given extra weight (e.g., 1.5x) due to its predictive power: **Fall Risk Index = Mean( Z_TUG + Z_5TSTS + Z_Risk_BBS + (1.5 * Z_FallHistory) )**. |
| **Postural Control** | • **Center of Pressure (CoP) Velocity** (on force plate) • **CoP Path Length** • **Limits of Stability (LoS)** • **BBS - Static Items** (e.g., single-leg stance, standing with eyes closed) | This construct focuses on the sensorimotor control of quiet and volitional posture. **CoP Velocity/Path Length** are direct, objective measures of sway during quiet standing. **LoS** assesses the ability to voluntarily displace weight. **BBS static items** provide clinical context. | **Method: Principal Component Analysis (PCA) Factor Score** 1. **Data Preparation:** Assemble the raw scores for all measures. Ensure polarity is consistent (higher = worse control for CoP measures; higher = better control for LoS and BBS). 2. **Run PCA:** Conduct a PCA on the correlation matrix of these measures. The first principal component (PC1) typically captures the common variance underlying "postural control." 3. **Extract Factor Scores:** Use the scoring coefficients from PC1 to compute a **Postural Control Factor Score** for each participant. This statistically derived score is an optimal linear combination of the original measures, creating a highly reliable single metric. If PC1 is not clear, a Z-score composite (as above) can be used as a robust alternative. |
